# Tracking invasions of a destructive defoliator, the gypsy moth (Erebidae: *Lymantria dispar*): Population structure, origin of intercepted specimens, and Asian introgression into North America

**DOI:** 10.1111/eva.12962

**Published:** 2020-04-15

**Authors:** Yunke Wu, Steven M. Bogdanowicz, Jose A. Andres, Kendra A. Vieira, Baode Wang, Allard Cossé, Scott E. Pfister

**Affiliations:** ^1^ Department of Ecology and Evolutionary Biology Cornell University Ithaca NY USA; ^2^ United States Department of Agriculture APHIS, PPQ, S&T, Otis Laboratory Buzzards Bay MA USA

**Keywords:** admixture zone, amplicon sequencing, Asian gypsy moths, assignment test, Invasive species, natural hybrids

## Abstract

Genetic data can help elucidate the dynamics of biological invasions, which are fueled by the constant expansion of international trade. The introduction of European gypsy moth (*Lymantria dispar dispar*) into North America is a classic example of human‐aided invasion that has caused tremendous damage to North American temperate forests. Recently, the even more destructive Asian gypsy moth (mainly *L. d. asiatica* and *L. d. japonica*) has been intercepted in North America, mostly transported by cargo ships. To track invasion pathways, we developed a diagnostic panel of 60 DNA loci (55 nuclear and 5 mitochondrial) to characterize worldwide genetic differentiation within *L. dispar* and its sister species *L. umbrosa*. Hierarchical analyses supported strong differentiation and recovered five geographic groups that correspond to (1) North America, (2) Europe plus North Africa and Middle East, (3) the Urals, Central Asia, and Russian Siberia, (4) continental East Asia, and (5) the Japanese islands. Interestingly, *L. umbrosa* was grouped with *L. d. japonica*, and the introduced North American population exhibits remarkable distinctiveness from contemporary European counterparts. Each geographic group, except for North America, shows additional lower‐level structures when analyzed individually, which provided the basis for inference of the origin of invasive specimens. Two assignment approaches consistently identified a coastal area of continental East Asia as the major source for Asian invasion during 2014–2015, with Japan being another source. By analyzing simulation and laboratory crosses, we further provided evidence for the occurrence of natural Asian–North American hybrids in the Pacific Northwest, raising concerns for introgression of Asian alleles that may accelerate range expansion of gypsy moth in North America. Our study demonstrates how genetic data contribute to bio‐surveillance of invasive species with results that can inform regulatory management and reduce the frequency of trade‐associated invasions.

## 1 INTRODUCTION

Biological invasions pose significant threats to native biodiversity and the global economy (Clavero & Garcia‐Berthou, 2005; Pimentel, Lach, Zuniga, & Morrison, 2000; Simberloff et al., 2013). Preventing or at least minimizing introductions of invasive species remains a major challenge for pest management programs (Courchamp et al., 2017). Design of effective preventative strategies can be facilitated by identifying routes of introduction, particularly the geographic origin of the invasion (Estoup & Guillemaud, 2010). Historical records sometimes provide direct evidence of how and from where invasive pests arrived, but such records have become less available with the complexity of modern trades and transportations. Alternatively, indirect methods based on genetic similarity between invasive populations and populations from the organism's native range allow such inference (Dlugosch & Parker, 2008). For example, if the invading population shows a close genetic relationship with a particular native population, the latter is often considered the origin of the invasion (Darling, Bagley, Roman, Tepolt, & Geller, 2008; Estoup & Guillemaud, 2010; Rosenthal, Ramakrishnan, & Cruzan, 2008). A successful inference draws on the premises that genetic differences exist among native populations and such differentiation can be detected by a set of genetic markers (Cornuet, Piry, Luikart, Estoup, & Solignac, 1999; Picq et al., 2017; Roe et al., 2018).

The gypsy moth *Lymantria dispar* (L.) is one of the most destructive invasive pests, ranking third among the most costly invasive insects in the world (Bradshaw et al., 2016). Its native range encompasses the temperate forests of Europe and Asia (Pogue & Schaefer, 2007). Although adult gypsy moths do not feed, the caterpillars are notoriously polyphagous, feeding on a wide variety of host plants belonging to 400–600 species (APHIS, 2016; Liebhold, Gottschalk, et al., 1995). During gypsy moth outbreaks, host plants can be completely defoliated and killed, causing shifts in natural forest composition as a result of host tree and seedling mortality (APHIS, 2016; Campbell & Sloan, 1977; Gottschalk, 1990). Three subspecies of gypsy moth are currently recognized, broadly corresponding to their geographic regions of origin: *L. d. dispar* (L.) mainly from Europe and introduced to North America, *L. d. asiatica* (Vnukovskij) from Asia east of the Ural Mountains, and *L. d. japonica* (Motschulsky) restricted to the Japanese archipelago (Pogue & Schaefer, 2007).

The subspecies *L. d. dispar*, also known as European gypsy moth (EGM), was introduced to the United States (U.S.) by a French artist and naturalist, Étienne Léopold Trouvelot, presumably from France between 1868 and 1869 (Forbush & Fernald, 1896). After a decade of remaining undetected, the population experienced successive outbreaks and has since invaded the northeastern U.S. and the Canadian Maritimes (Liebhold, MacDonald, Bergdahl, & Mastro, 1995). As one would expect from the introduction history, some studies identified an intermingled genetic pattern among populations from North America and Europe (Picq et al., 2017; Zahiri, Schmidt, Schintlmeister, Yakovlev, & Rindos, 2019). Alternatively, other studies revealed significant distinctiveness between the North American population and contemporary European populations (Wu et al., 2015; Zhang et al., 2019). If the latter scenario is supported, it may suggest that the North American population is on the evolutionary trajectory of becoming a lineage independent from its source population(s).

**FIGURE 1:**
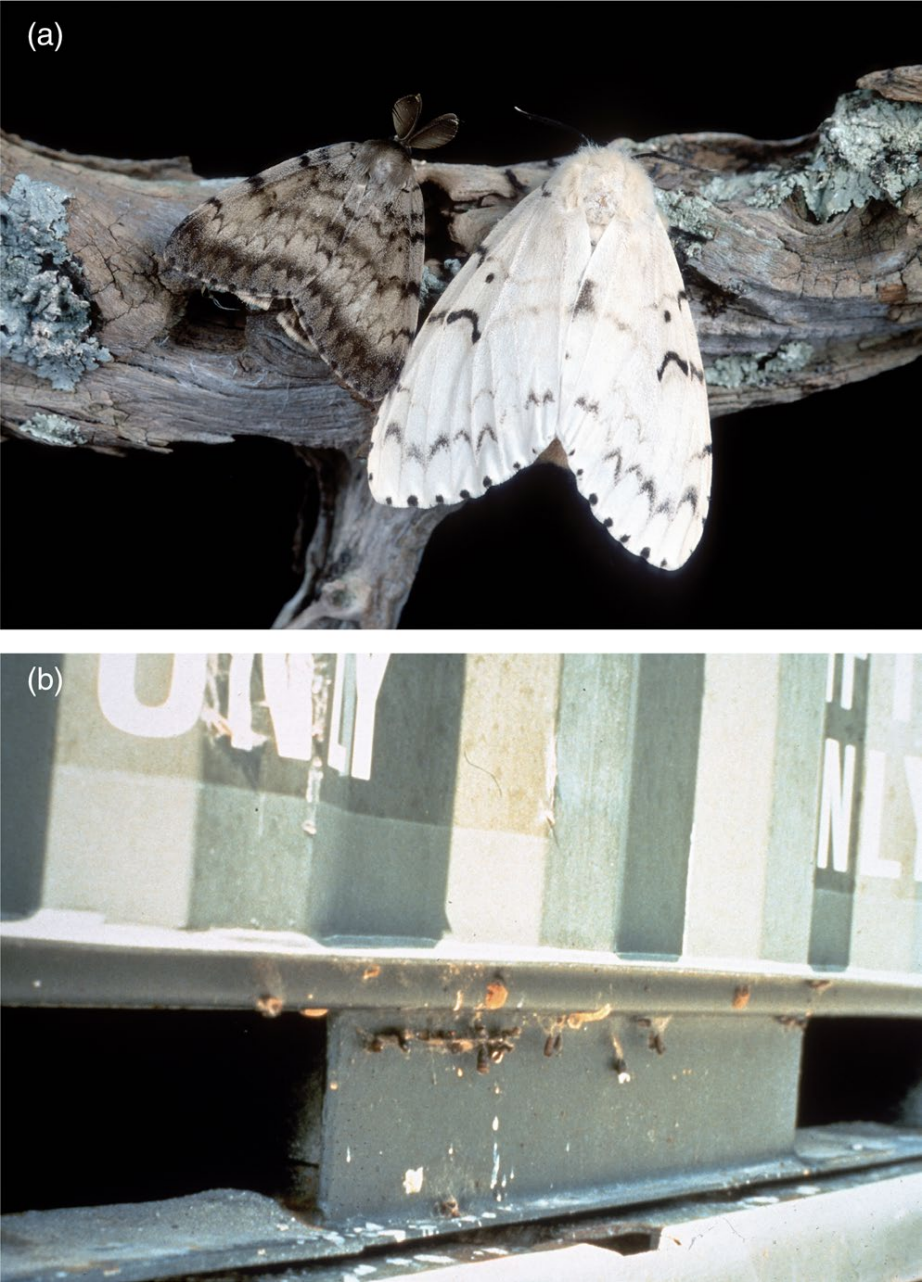
(a) Adult *Lymantria dispar* exhibiting sexual dimorphism. Male moths on the left and female moths on the right. (b) Cargo containers arriving at U.S. ports of entry with *L. dispar* egg masses and pupal cases attached. (Photos taken from Otis Laboratory Archive)

After the initial invasion of EGM, the U.S. and Canada have undertaken tremendous efforts to prevent subsequent introductions of the Asian gypsy moth (AGM), a collective term referring to subspecies *L. d. asiatica* and *L. d. japonica* as well as three closely related species once considered subspecies of gypsy moth (APHIS, 2016; Bogdanowicz, Schaefer, & Harrison, 2000; Pogue & Schaefer, 2007). The most notable difference between AGM and EGM is that AGM females are capable of sustained ascending flight over several kilometers before oviposition (Yang et al., 2012). This characteristic contrasts with the incapability of flight in most EGM females including the North American population, which usually oviposit near the site of pupation (Pogue & Schaefer, 2007). Asian gypsy moth also has different host preferences and exhibits elevated levels of polyphagy compared with EGM (Baranchikov, 1989). Thus, if established, AGM populations can potentially spread faster and further, while consuming plant species currently unaffected by EGM. The introduction of AGM in North America may also result in introgressive hybridization of flight and host preference alleles that can be partially inherited in the hybrid progenies, which could accelerate the spread of future outbreaks (Keena, Grinberg, & Wallner, 2007). Since the 1990s, AGM egg masses (rarely pupae) attached to surfaces of cargo and on ship superstructures have been intercepted annually at ports of entry in North America (APHIS, 2016; Savotikov, Smetnik, & Orlinskii, 1995; Figure 1).

Tracking invasions of gypsy moths depend on how populations are structured across native and invasive ranges. Multiple genetic groups have been recognized within *L. dispar*, some of which are connected by gene flow (Bogdanowicz, Mastro, Prasher, & Harrison, 1997; deWaard et al., 2010; Keena, Côté, Grinberg, & Wallner, 2008; Wu et al., 2015; Zahiri et al., 2019; Zhao, Wu, Kurenshchikov, Ilyinykh, & Shi, 2019). However, the complex evolutionary history of gypsy moth, further confounded by human‐aided movement, cannot be delineated by a small number of nuclear or mitochondrial markers, which in turns limits the capacity to determine the origin of invasion. Larger genetic datasets, on the other hand, are more likely to resolve population structuring at both regional and local scales. A recent proof‐of‐concept study used single nucleotide polymorphism (SNP) data to differentiate eight laboratory‐reared *L. dispar* colonies (Picq et al., 2017). Based on the observed differentiation, the study demonstrated the potential for genomic data to assign individuals to their source populations. However, because the genetic diversity of colony moths is biased due to excessive, long‐term inbreeding, it remains unclear the extent to which the study applies to wild populations (Picq et al., 2017).

In this study, we have generated a panel of 60 genetic markers (55 nuclear and 5 mitochondrial) derived from natural populations. We used this panel to genotype over one thousand gypsy moth specimens sampled across Asia, Europe, North America, and North Africa, representing the largest dataset of field‐collected gypsy moth to date. We supplemented the above sampling with *L. umbrosa* collected from Hokkaido, Japan, due to its possible genomic admixture with *L. d. japonica* (Pogue & Schaefer, 2007; Zhang et al., 2019). We characterized genetic differentiation for a better understanding of evolutionary relationships between species, subspecies, and populations. Based on the recovered geographic structuring, we further investigate the ancestry of AGM intercepted at U.S. ports of entry and evidence of Asian introgression into North America. Two different approaches, the discriminant analysis of principal components (DAPC; Jombart, Devillard, & Balloux, 2010) and the support vector machine predictive (*svm*) model (Chen et al., 2018), were used for the assignment analyses. Our application provides critical information for the development of effective management measures, helps monitor and prevent additional AGM introductions, and ultimately facilitates safer bilateral trade.

## 2 MATERIALS AND METHODS

### 2.1 Sample collection and DNA extraction

**FIGURE 2:**
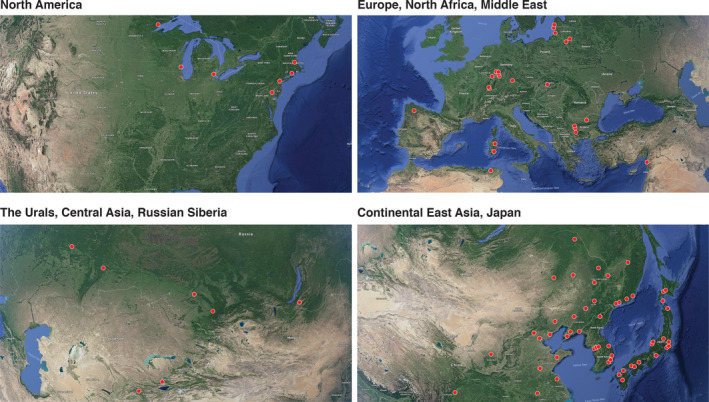
Collection sites of wild populations of *Lymantria dispar* throughout the Holarctic region. Two populations of *L. umbrosa* were collected from Hokkaido, Japan

**FIGURE 3:**
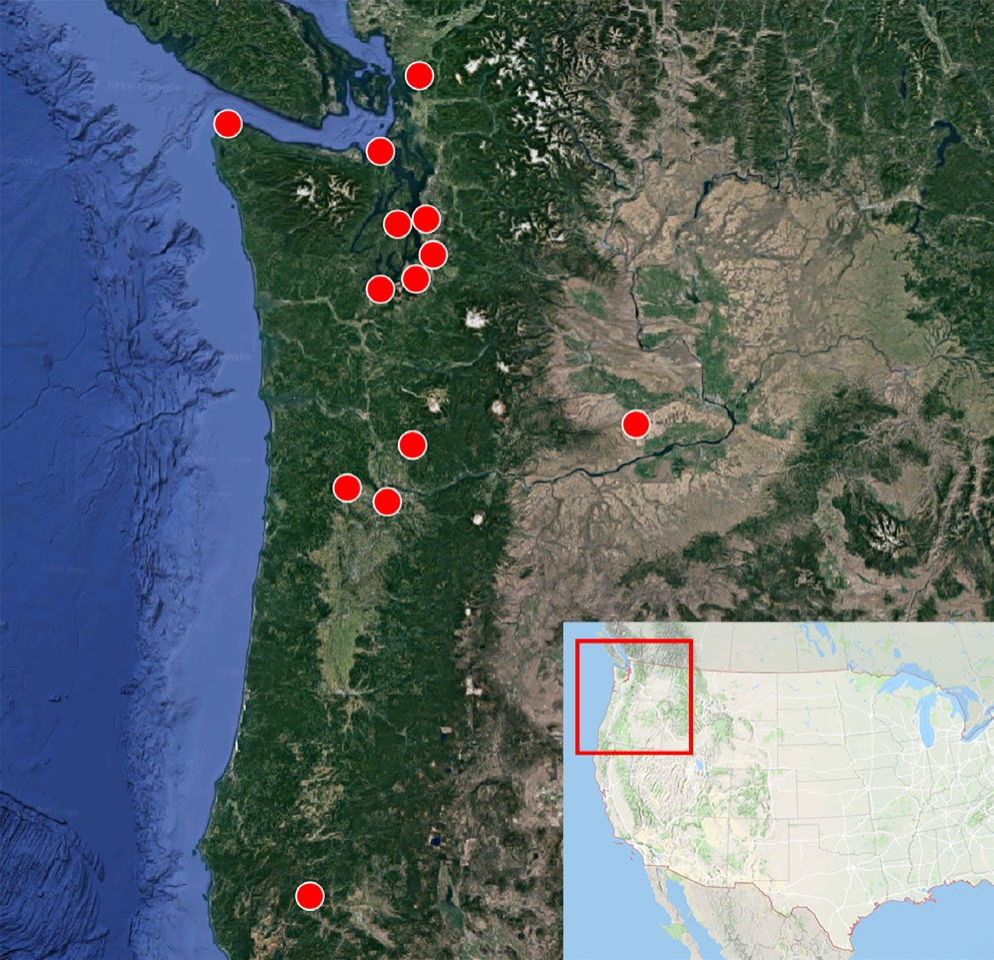
Collection sites for 37 male moths intercepted in the Pacific Northwest between 2014 and 2015. Four of those moths were initially identified as Asian gypsy moth by USDA

We covered as much of the geographic distribution of *Lymantria dispar* as possible, with an intense sampling effort in East Asia and Europe. Gypsy moth samples (*n* = 1,181) were collected in 17 countries at 89 different geographic locations (average of 37 specimens/locality) (Figure 2, Table S1). Adult moths were caught in traps baited with disparlure as the sex‐attractant pheromone. Specimens of *L. umbrosa* (*n* = 37) were collected from Hokkaido and morphologically identified (Hannah Nadel). We included *L. umbrosa* in all subsequent analyses given its sister status with *L. dispar* and potential introgression into the latter species (Zhang et al., 2019). We further genotyped egg masses intercepted at U.S. ports of entry (Table S3) and male moths trapped in the Pacific Northwest between 2014 and 2015 (Figure 3, Table S4). Those moths primarily comprised individuals diagnosed as EGM by U.S. Department of Agriculture (USDA) plus four specimens diagnosed as AGM. Specimens were stored dry either at −20°C or −70°C. Genomic DNA was extracted using the Qiagen DNeasy Blood & Tissue Kit (Qiagen, Venlo, the Netherlands) following the manufacture's protocol or a modified Proteinase K protocol (Maniatis, Fritsch, & Sambrook, 1982). For the latter protocol, a moth leg or antenna was placed in 500 µl extraction buffer (5% Proteinase K, 0.1% Tergitol, 1X TE buffer) and incubated at 37°C overnight. The extraction was deactivated by heating at 75°C for 30 min and then stored at −20°C.

### 2.2 Genome‐wide genetic marker discovery

To identify polymorphic amplicons while reducing ascertainment bias, we first selected 12 individuals (two per locality) from a broad geographic range including Germany, Japan, China, South Korea, the Russian Far East, and U.S. For each of these samples, we generated a double‐digest restriction site‐associated DNA (ddRAD) library (Peterson, Weber, Kay, Fisher, & Hoekstra, 2012), cutting with *SbfI* and *MspI* while simultaneously ligating P1 (*SbfI*) and P2 (*MspI*) adaptors to the fragmented DNA. Following amplification, the 12 samples were pooled and double size‐selected (targeted range: 350–550 bp) using AMPure XP beads. This size‐selected library was then sequenced using an Illumina MiSeq platform with paired‐end reads (2 × 300 bp) at the Cornell University's BioResource Center.

After an initial quality check using FastQC (Andrews, 2010), raw Illumina reads were assembled into contigs (unique consensus sequences from multiple Illumina reads) using NGen (v.11) and default parameters. The resulting contigs (*n* = 1,713) were then filtered and the following were discarded: (a) contigs with low coverage (<20X) to reduce sequencing error bias; (b) contigs with extremely high coverage (>10,000X) to avoid repetitive regions in the genome; and (c) monomorphic contigs to exclude uninformative regions. After applying these filters, 117 contigs associated with multiple SNPs were initially selected. Primer pairs were developed to amplify a fragment of each selected contig using BatchPrimer3 (You et al., 2008; for primers sequences, see Table S2). All amplicons, or called loci hereinafter, were constrained to between 350–425 bp in size. Primers were tested through individual PCRs with a sub‐sample of gypsy moth populations. Sixty loci showing robust amplification (*n*_nuclear_ = 55 and *n*_mitochondrial_ = 5) were kept in the final genotyping panel. The median number of SNP per locus is 42 (25% quantile = 30, 75% quantile = 57). Sequence variants detected by a custom Perl script (available upon request) defined alleles at those loci for the individuals genotyped.

### 2.3 Individual genotyping

Individual moths were genotyped at 60 loci in four randomly divided multiplex PCRs using the QIAGEN Multiplex PCR Kit (Qiagen, Venlo, the Netherlands). Primers targeting mitochondrial loci were added at 0.5X concentration relative to the nuclear primers to account for the increased copy number of the mitochondrial genome. Multiplex PCRs were combined within samples and barcoded using Nextera S5 (*n* = 16) and N7 (*n* = 24) barcode primers, then cleaned with 0.7X AMPure XP beads. The resulting library (*n* = 384 individuals per library) was sequenced on an Illumina MiSeq with paired‐end reads (2 × 300 bp) at Cornell University's BioResource Center. For allele calls at each locus, we ran the custom Perl script to extract reads and assign them to the appropriate locus and individual. The script (a) trimmed adapters and low‐quality reads, (b) merged overlapping reads, (c) identified reads corresponding to each locus, (d) collapsed identical reads for each individual, and (e) identified the top two haplotypes for individuals at all loci (i.e., their diploid genotypes). We set a minimum read length of 225 bp and applied a matching command (‐x) that requires 90% of the first 40 bp of a read to align to and match a reference sequence at each locus, thereby minimizing PCR artifacts and paralogs. Specimens that had < 10X coverage across all loci or failed at more than 15 loci (>25% missing data) were excluded from subsequent analyses. The final worldwide dataset include 950 field‐collected *L. dispar* and 36 *L. umbrosa* specimens.

### 2.4 Population diversity analysis

Genetic diversity was estimated for nuclear loci for all populations of *L. dispar* and *L. umbrosa*. Mitochondrial loci were excluded because they were linked and heterozygosity indices did not apply. Summary statistics including number of polymorphic loci, average number of alleles per locus, mean observed and expected heterozygosity, and average gene diversity were calculated for each country or geographic region in Arlequin 3.5 (Excoffier & Lischer, 2010). Similarly, average number of private alleles per locus was computed using the R package *poppr* 2.8.2 (Kamvar, Tab ima, & Grünwald, 2014). We further assessed linkage disequilibrium between all pairs of nuclear loci using Genepop 4.7 (Rousset, 2008) with 10,000 dememorizations in one million Markov chain approximations. Pairwise fixation indices (*F*_ST_) were estimated in Genepop among genetic clusters identified below.

### 2.5 Population structure analyses

To characterize genetic differentiation among our samples, we used Bayesian and multivariate clustering methods. The Bayesian clustering analysis was performed in STRUCTURE 2.3.4 (Pritchard, Stephens, & Donnelly, 2000) with the admixture model, which allowed individuals to have mixed ancestry from multiple clusters. Only nuclear data were used due to the requirement of unlinked markers. We tested from *K* = 1 to *K* = 10 with 10 replicates for each value of *K*. Markov chain Monte Carlo was run for one million generations after a burn‐in of 100,000 generations. The best *K* value was selected using the Δ*K* method in STRUCTURE Harvester (Earl & vonHoldt, 2012; Evanno, Regnaut, & Goudet, 2005), and the associated replicates were aligned under the Greedy algorithm of CLUMP 1.1.2 (Jakobsson & Rosenberg, 2007). Mean individual and population matrix were plotted in DISTRUCT 1.1 (Rosenberg, 2004). We discarded individual membership coefficients with posterior probabilities < 0.05 and assigned corresponding values proportionally to other membership coefficients so that the total coefficients for each individual still summed to one. Following a similar approach for analyzing genetic structure that may exhibit hierarchies (Coulon et al., 2008), we repeated the analysis on each of the *K* groups inferred in the previous step. Individual run length was adjusted to 500,000 generations with 200,000 burn‐in period. This approach allows the identification of both higher and lower levels of population structure (Balkenhol et al., 2014).

A multivariate, hierarchical Discriminatory Analysis of Principal Components (DAPC) analysis was performed as follows based on combined nuclear and mitochondrial data. First, we used a hierarchical Ward algorithm through the function *find.clusters* to determine the minimum number of geographic groups that are genetically distinct from each other while best summarizing variations in the data. The number of retained principal components (PC) was optimized by cross‐validation starting with 300 PCs to avoid model over‐fitting. Second, to further explore lower‐level population structure, we repeated DAPC for individual geographic groups inferred in the previous step using a priori information of specimen's group membership. The optimal number of clusters per group was then chosen by varying *K* from 1 to 20. Lastly, after determining the total number of genetic clusters present among all groups, we carried out a final DAPC analysis with the full dataset and a priori information of specimen's cluster membership to display relationships among clusters. The analysis started with 300 PCs and was cross‐validated. All multivariate analyses were run on the devel version of R package *adegenet* 2.1.1 (Jombart et al., 2010).

### 2.6 Assignment of intercepted AGM

Asian gypsy moths intercepted in the U.S. (26 eggs masses and five adults) were assigned to a reference panel that consisted of populations from China, the Russian Far East, South Korea, and Japan. We selected this panel because the mitochondrial haplotype of intercepted individuals matched that of *L. dispar* from this region (Djoumad et al., 2017). The reference panel also included *L. umbrosa* due to (a) the recent confirmation of one adult *L. umbrosa* male in the state of Washington and (b) a possible mix‐up of records between *L. dispar* and *L. umbrosa* (USDA APHIS Pest Interception Database). Central Asian or Russian Siberian populations were not included as they possess EGM‐type mitochondrial genomes (Martemyanov et al., 2019; Zahiri et al., 2019; Zhao et al., 2019). A total of 84 individual eggs were sampled (2–8 eggs per egg mass).

Two types of assignment tests were performed using the predictive model in DAPC and the *svm* predictive model implemented in the R package *assignPOP* 1.1.5 (Chen et al., 2018). To measure conclusiveness in the assignment, we adopted the “relative probability” following Schmidt et al. (2019), which was defined as the ratio between the highest and the second highest posterior probability of membership for a given specimen. A specimen was only assigned to a given reference group if the relative probability was ≥ 2, otherwise it was considered inconclusive. Congruence between the two types of tests would indicate a robust assignment.

Before the assignment test, diagnostic power of the reference data was assessed through resampling validation. For validation in DAPC, 10 individuals were randomly selected from each reference group to form a test set, and the remaining specimens were used as the training set. A total of 20 replicates were performed with a different random test set each time. When test individuals were correctly assigned back to their original population, we proceeded to predict geographic origins of intercepted AGM specimens by the function *predict.dapc*. For validation in *assignPOP*, the Monte Carlo procedure randomly selected 10% individuals from each reference group as the testing data and the remaining 90% as the training data, which was crossed by top half loci with highest *F*_ST_ values and all 60 loci. We ran 50 replicates for each cross‐validation under the *svm* model retaining the same number of PCs as in DAPC. Then, assignment test was performed using the function *assign.X*.

### 2.7 Detection of ongoing hybridization

When AGM evades inspection at ports of entry, it may hybridize with the North American population. Evidence for ongoing hybridization can be demonstrated by detecting early‐generation progenies (i.e., F1, F2, and backcrosses). Here, we focused on the Pacific Northwest because this region is not only a likely hotspot for AGM introduction, but also witnessed recurrent introduction of EGM transported by humans from its established range on the Atlantic Coast. We examined 37 male gypsy moths captured in Washington and Oregon during 2014 and 2015 for their identity as AGM, EGM, or hybrids based on posterior probability of membership. Among them, four moths had been previously identified as AGM and the remaining 33 as EGM by the USDA standard gypsy moth assay (available upon request), which does not test for hybrids.

References for hybrid assignment were established through two approaches. First, we randomly selected 100 specimens collected from East Asia (including *L. umbrosa*) to represent the AGM parent and another random 100 U.S. specimens as the EGM parent. First generation (F1) and second generation (F2 and backcrosses between F1 and EGM) progenies were simulated for 100 individuals for each progeny group using the function *hybridize* in *adegenet*. For demonstration purposes, we did not simulate further generations. Field‐collected parents and simulated hybrids were all used as reference. Second, because hybridization between geographic races of *L. dispar* can generate abnormal sex ratios or intersex progenies (Goldschmidt, 1934), which may violate the assumption from standard hybridization simulation, we performed actual laboratory hybridization using reared colony moths to complement our computer simulations. Moth colonies were maintained at the Otis Laboratory under USDA quarantine facility permits and selected based on availability during the experimental design. We obtained F1 progenies by crossing *L. d. japonica* originating from northern Iwate District, Japan (collected in 2005) and *L. d. dispar* from New Jersey, U.S (collected in 1967). Sixteen mating pairs (8 pairs of AGM ♂ × EGM ♀ and 8 pairs of EGM ♂ × AGM ♀) were set up and F1 individuals were reared and randomly sampled. Due to the prolonged generation time of *L. dispar*, we did not perform crossing experiments for the second generation. The final laboratory reference data included 11 AGM parents, 14 EGM parents, and 249 F1 progenies. Although this dataset only represented a subset of AGM and possible hybrids, it was reasonable to assume that other crossings (e.g., U.S. × China or U.S. × Korea) would produce similar results, because divergence between AGM and the North American population was much greater than that within AGM (see Results). Results from the laboratory dataset can help assess the robustness of the simulation and validate our conclusion. Similar to the assignment tests of intercepted AGM specimens, both DAPC and *assignPOP* were used for reference cross‐validation (20 and 50 replications, respectively) and subsequent assignment of Washington and Oregon specimens.

## 3 RESULTS

### 3.1 Genetic diversity among worldwide populations

Linkage disequilibrium was only found in 5% of all pairwise comparisons from the 55 nuclear loci after correcting for multiple comparisons, suggesting that they are mostly unlinked. Population diversity statistics are summarized in Table 1. Despite variation in number of specimens sampled from Asia, Europe, and North America, the numbers of polymorphic loci were similar across countries or regions (mean = 50), but loci that were invariable within each country or region were different. The mean number of alleles per locus and private allele per locus were much greater in East Asia than other populations (two tailed *t* test, *t* (18) = 10.13, *p* < .0001; *t* (18) = 6.56, *p* < .0001). Average genetic diversity and heterozygosity were also highest in East Asia.

**TABLE 1:** Sampling countries and regions, number of sampled individuals, and summary population diversity indices based on nuclear data. For detailed sampling localities in each country or region, see Table S1

Country and Region	*N*	*n*	P	A_N_	P_R_	D	H_O_	H_E_
*Lymantria dispar*								
**North America**								
United States	7	98	50	7.4	3.2	0.402	0.449	0.426
**Europe, North Africa, Middle East**								
Bulgaria	4	22	51	8.2	1.9	0.578	0.628	0.620
Slovakia	1	12	53	4.9	0.5	0.571	0.605	0.584
Lithuania	3	22	50	7.2	0.9	0.609	0.638	0.636
Latvia	2	15	53	6.2	0.6	0.602	0.627	0.630
Germany	7	79	52	15.6	6.3	0.595	0.642	0.646
France	2	23	53	7.3	1.2	0.566	0.598	0.587
Italy	2	22	49	7.3	2.1	0.466	0.561	0.533
Spain	1	12	46	4.0	0.7	0.420	0.448	0.423
Tunisia	1	9	42	2.7	0.5	0.327	0.412	0.337
Syria	1	12	51	4.6	1.6	0.492	0.534	0.509
**The Urals, Central Asia, Russian Siberia**								
Urals (Russia)	2	20	50	7.6	1.2	0.625	0.701	0.677
Kyrgyzstan	1	10	50	3.8	0.4	0.525	0.585	0.545
Kazakhstan	1	5	49	4.0	0.4	0.616	0.652	0.640
Russian Siberia	3	23	50	7.8	1.2	0.622	0.635	0.662
**East Asia**								
Main China^a^	16	131	53	35.1	15.1	0.712	0.726	0.761
Northeastern China	7	77	46	22.5	8.2	0.700	0.764	0.791
Russian Far East	5	52	52	21.8	4.7	0.715	0.754	0.777
South Korea	7	136	52	34.6	11.0	0.727	0.742	0.789
Japan	16	170	47	38.8	19.0	0.698	0.718	0.761
*Lymantria umbrosa*								
Japan	2	36	50	13.5	2.7	0.684	0.762	0.751

Note

*N*, number of collecting sites in each country or region; *n*, total number of individuals sampled in each country or region; P, number of polymorphic loci. Each locus is defined by a primer pair and contains multiple SNPs; A_N_, mean number of alleles per locus; P_R_, mean number of private alleles per locus; D, average gene diversity over loci; H_E_, mean expected heterozygosity over loci; H_O_, mean observed heterozygosity over loci.

^a^ Main China is defined as areas west of the Changbai Mountains; northeastern China is east of the Changbai Mountains.

### 3.2 Population structure of gypsy moth

**FIGURE 4:**
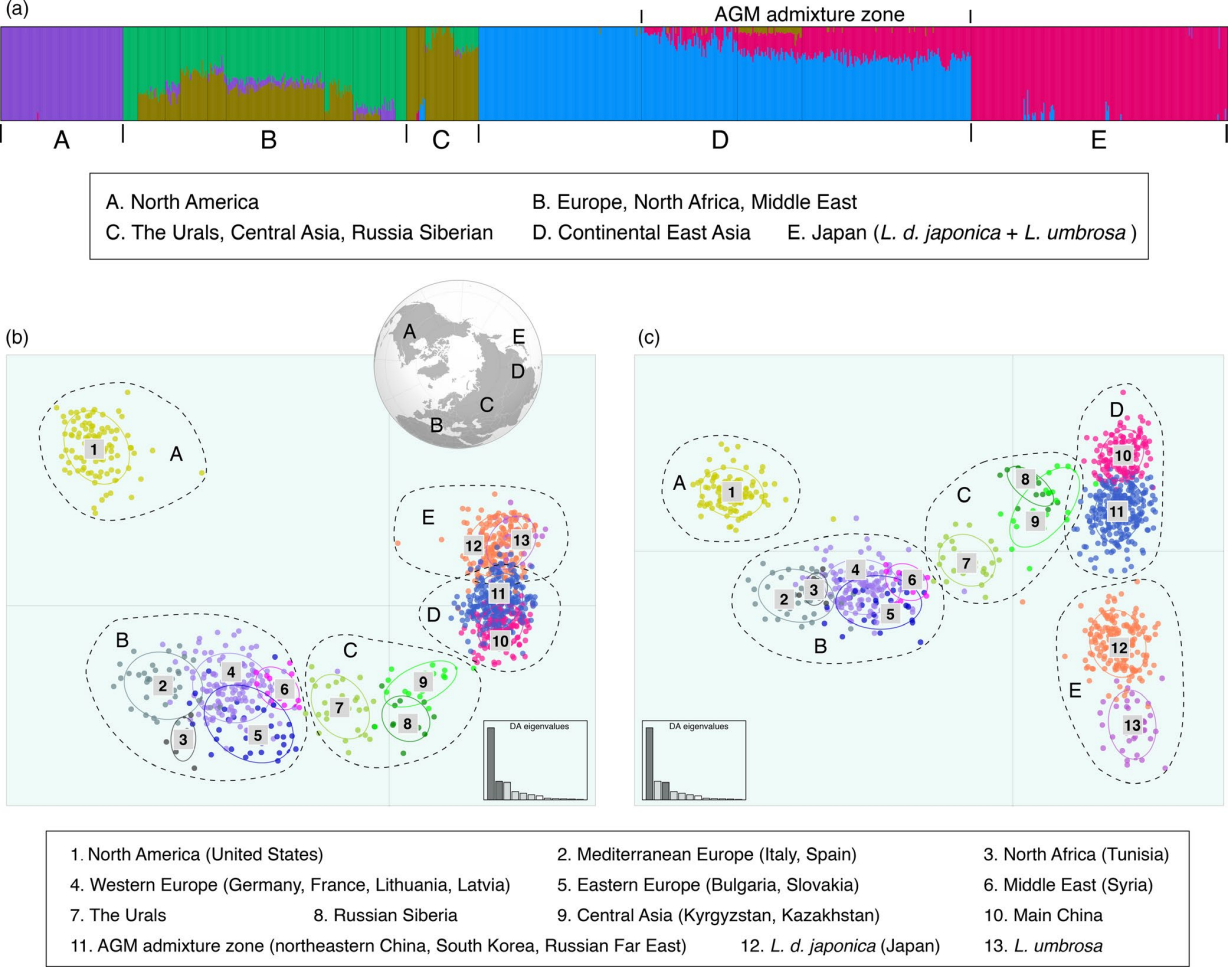
Global population structure of *Lymantria dispar* and *L. umbrosa*. (a) STRUCTURE barplot for *K* = 5 based on nuclear data only. The five geographic groups were labeled A–E and followed a west‐to‐east longitudinal gradient. (b) DAPC scatterplot based on both nuclear and mitochondrial data for discriminant function 1 and 2. Insert shows the five geographic groups on the Northern Hemisphere when viewed from above the North Pole. (c) DAPC scatterplot result for discriminant function 1 and 3. Number 1–13 correspond to genetic clusters identified within the five geographic groups (A–E)

The Δ*K* method selected *K* = 5 for STRUCTURE runs based on nuclear data only (Figure S1). The worldwide populations of *L. dispar* and *L. umbrosa* were separated into five geographic groups (Figure 4a): (1) North America (group A), (2) Europe plus neighboring North Africa and Middle East (group B), (3) the Urals, Central Asia, and Russian Siberia (group C), (4) continental East Asia (group D), and (5) Japan (group E). The North American population was distinct from EGM sampled in the native range. Interestingly, *L. umbrosa* did not form its own group but instead was grouped with *L. d. japonica*. Genetic admixture was observed in Europe and continental East Asia. The latter area, where specimens can attribute a minor portion of their ancestry to moths from Japan, encompassed northeastern China (east of the Changbai Mountains), South Korea, and the Russian Far East. We designated this area as the AGM admixture zone. Individual STRUCTURE analysis for each of the inferred geographic groups identified lower‐level population structures except in North America (Figure S2). Group B was subdivided into two clusters: the Mediterranean region (Italy, Spain, Tunisia, Syria, and a few French specimens) and other European populations. Within group C, each of the three geographic areas was recovered as a distinct genetic cluster. Group D also subdivided into two clusters: the AGM admixture zone and remaining Chinese populations. In the Japanese group E, *L. d. japonica* and *L. umbrosa* were resolved as two genetic clusters.

For population structure using DAPC, the hierarchical Ward algorithm did not determine a single optimum number of genetic clusters and found equal support for models from a range of approximately *K* = 5 to *K* = 15 (50 PCs retained, Figure S3). The *K* = 5 model recovered the same groups recovered by the STRUCTURE analysis. We re‐ran the analysis without the mitochondrial loci, and the result was nearly identical (Figure S4). We then repeated the DAPC analysis for each of the five geographic groups to explore lower‐level structure. The BIC scores and structure‐like posterior probability of membership for individual specimens are shown in Figure S5. While the North America population did not support further partitioning, the other four groups can be divided into 2–5 genetic clusters, either identical or congruent with results from individual STRUCTURE analysis. Notably, group B was further split into North Africa (Tunisia), Middle East (Syria), and three European clusters (eastern, western, and Mediterranean). The two Japanese taxa, *L. d. japonica* and *L. umbrosa*, also can be distinguished by DAPC when analyzed alone. However, if mitochondrial loci were excluded, DAPC again supported a single Japanese population (Figure S5). Relationships among the 13 genetic clusters were displayed through a final run of DAPC retaining 57 PCs. Based on the first two discriminant functions, genetic positions of those clusters largely resembled their geographic positions across the Northern Hemisphere as if viewed from above the North Pole (Figure 4b). The North American population appears remarkably isolated from other EGM. Populations from East Asia overlapped along the first two discriminant functions but showed clear separation along the third one (Figure 4c). We further calculated pairwise nuclear *F*_ST_ values between clusters (Table 2). Although the North American population most likely originated from Western Europe, their *F*_ST_ value was comparable to or even greater than those derived from some EGM‐AGM comparisons or between *L. d. japonica* and *L. umbrosa*.

**TABLE 2:** Pairwise nuclear *F*_ST_ values of among 13 genetic clusters identified by DAPC (n: number of specimens)

	Genetic clusters	*n*	1	2	3	4	5	6	7	8	9	10	11	12
1	North America	98												
2	Eastern Europe	34	0.158											
3	Western Europe	139	0.135	0.020										
4	Mediterranean Europe	34	0.160	0.054	0.066									
5	Middle East	12	0.277	0.122	0.136	0.157								
6	North Africa	9	0.304	0.153	0.145	0.107	0.301							
7	Urals (Russia)	20	0.256	0.091	0.068	0.199	0.210	0.285						
8	Russian Siberia	23	0.282	0.127	0.104	0.241	0.238	0.320	0.055					
9	Central Asia	15	0.369	0.213	0.178	0.323	0.306	0.403	0.148	0.115				
10	Main China	131	0.261	0.139	0.130	0.213	0.198	0.268	0.089	0.086	0.108			
11	AGM admixture zone^a^	265	0.229	0.126	0.112	0.196	0.184	0.246	0.077	0.075	0.097	0.017		
12	Japan	170	0.252	0.157	0.136	0.215	0.206	0.258	0.122	0.137	0.153	0.108	0.057	
13	*Lymantria umbrosa*	36	0.292	0.165	0.145	0.238	0.219	0.286	0.127	0.138	0.161	0.089	0.048	0.024

^a^ The AGM admixture zone comprises northeastern China, the Russian Far East, and the Korean Peninsula.

### 3.3 Geographic origin of intercepted AGM

**FIGURE 5:**
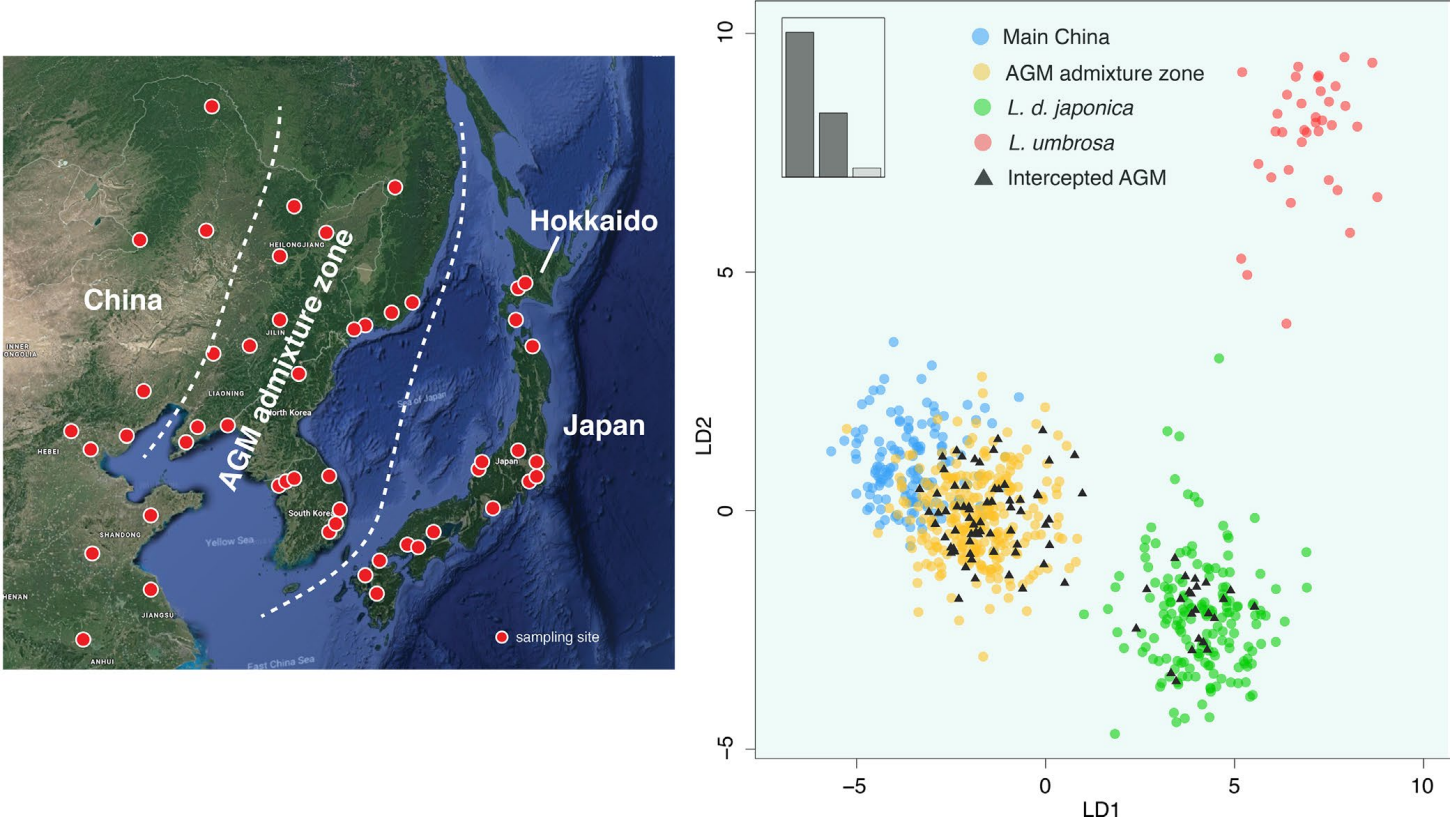
Assignment of Asian gypsy moths intercepted in the U.S. between 2014 and 2015. (a) Map showing the AGM admixture zone between main China and Japan. (b) DAPC scatterplot result with inferred assignment of intercepted individual AGM eggs or adults (black triangles)

Based on results from the DAPC analysis, we selected four East Asian clusters as reference for the assignment test (Figure 5a): main China, the AGM admixture zone, *L. d. japonica* and *L. umbrosa*, using nuclear and mitochondrial loci. Diagnostic power of the dataset was validated through resampling the reference. In DAPC, 20 random replicates showed very high assignment success with an average overall accuracy of 96.3% (92.5%–100%). Incorrect assignment of test specimens from main China to the AGM admixture cluster accounted for all errors, an expected result given their partial overlap in DAPC. For cross‐validation in *assignPOP*, using the top half loci with highest *F*_ST_ values versus. all 60 loci produced similar overall assignment accuracy (93.4% and 93.5%). Per cluster accuracy ranged between 82.0% and 99.8%.

The two types of test, DAPC and *assignPOP* (PC = 26), produced nearly identical assignments for 84 AGM eggs and five adults. All specimens but one were decisively assigned (relative probability ≥ 2) by the former test and all decisively by the latter test. For the only inconclusive specimen (relative probability < 2) in DAPC, its most likely origin was the same cluster that received the decisive assignment by *assignPOP*. Since multiple eggs were sampled from the same egg mass, those sibling eggs should be assigned to the same reference. In our case, all but one egg mass had identical or at least congruent assignment (i.e., one egg assigned and one egg inconclusive) among sibling eggs. The only exception, which was identified by both tests, produced a conflicting result that assigned one egg to the AGM admixture zone and other three eggs to *L. d. japonica*. This could be due to mislabeling or contamination. Of the 26 AGM egg masses, 21 (80.8%) were assigned to the AGM admixture zone and four (19.0%) to *L. d. japonica* (Figure 5b; Table S3). The percentage was similar for AGM adults, with four of five moths originated from the AGM admixture zone and the fifth moth from Japan. No egg mass or adult was assigned to either the main China cluster or *L. umbrosa*.

### 3.4 Detection of ongoing Hybridization in the Pacific Northwest

**FIGURE 6:**
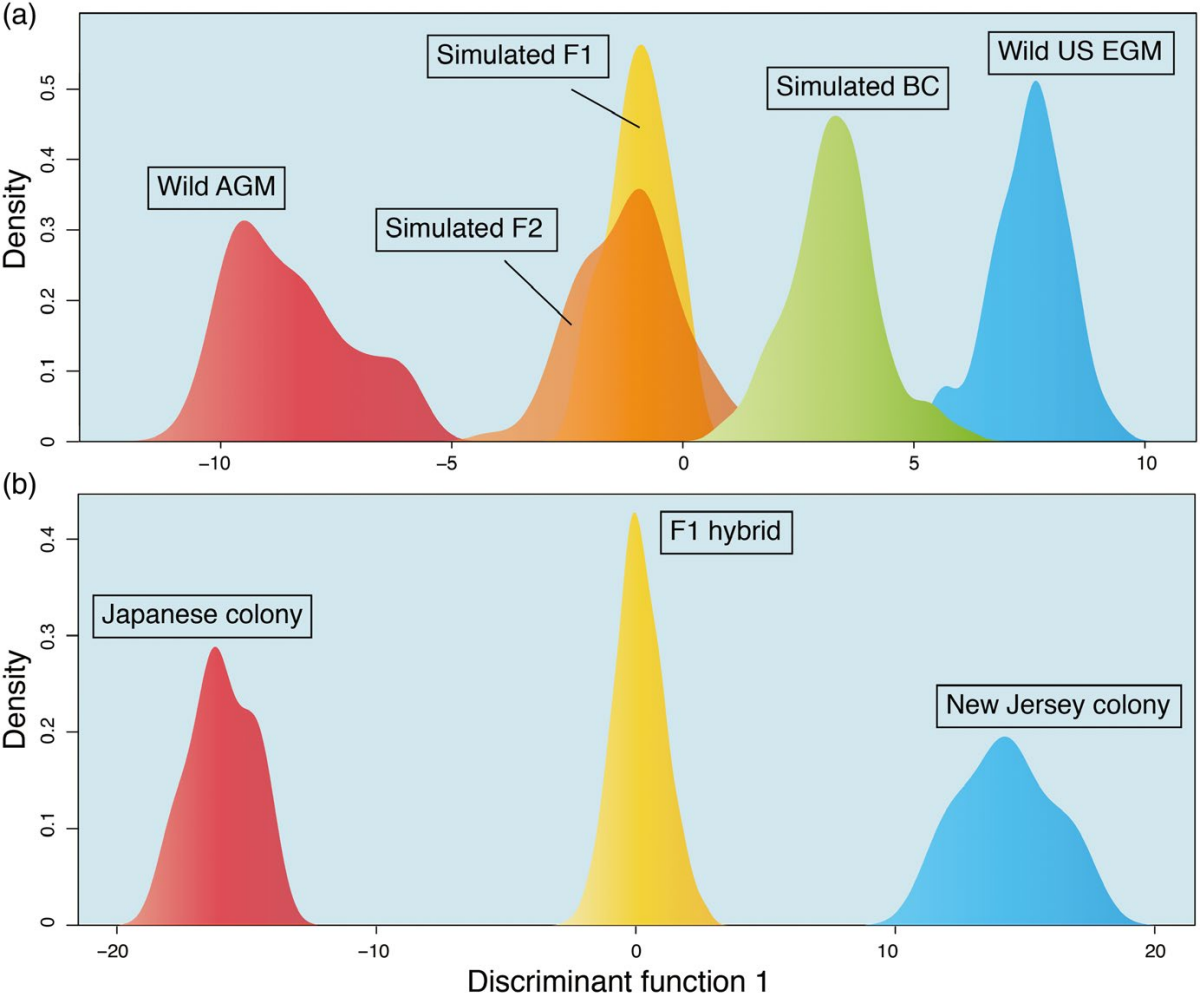
Histogram showing densities of individuals on the first discriminant function of DAPC. (a) The simulated dataset. The F1, F2, and backcross (BC) progenies were simulated using wild‐collected parents. (b) The laboratory dataset. The F1 progenies were produced by crossing a Japanese colony with a New Jersey colony

Cross‐validation for the simulated dataset demonstrated that AGM and EGM parents can be easily distinguished from first and second generation hybrids in either DAPC or *assignPOP* (PC = 10, Figure 6a). Assignment accuracy for the parents ranged between 98.0% and 100%. Both tests also readily identified F1‐EGM backcrosses (accuracy > 96.0%). However, about half of the time neither method was able to differentiate between F1 and F2 progenies. This was not unexpected since genomic profiles can be similar between those two groups given the number of markers used. For the laboratory dataset, F1 progenies seemed to follow Mendelian inheritance and resembled those generated by simulation (Figure 6b). Cross‐validation using DAPC and *assignPOP* (PC = 13) showed perfect assignment of resampled parents and F1 with either top half high *F*_ST_ loci or the full set of loci.

Among the 37 *L. dispar* adult males trapped in Washington and Oregon, the simulated dataset confirmed four AGM and 15 EGM specimens as pure parents. Both DAPC and *assignPOP* yielded identical assignment for those 19 moths (Table 3, Table S4). However, as many as 13 moths were likely backcrosses between F1 and EGM. Additionally, a few specimens (3 by DAPC and 5 by *assignPOP*) had their combined probability as pure AGM or EGM less than 0.05, but they could not be decisively assigned to either F1, F2 or backcross. Those individuals were considered as “undetermined hybrids.” When using the laboratory dataset, DAPC and *assignPOP* identified at least 5 individual as hybrids. Those five specimens were also undoubtedly identified as hybrids under the simulated dataset (Table S4).

**TABLE 3:** Assignment of 37 adult gypsy moth trapped in Washington and Oregon states in 2014–2015 based on the simulated and laboratory datasets

	Simulated hybrids							Laboratory hybrids			
	AGM	EGM	F1	F2	BC	UH	Inconclusive	AGM	EGM	F1	Inconclusive
DAPC	4	15	0	0	13	3	2	4	28	5	0
*assignPOP*	4	15	0	0	7	5	6	2	21	8	6

Note

*Lymantria umbrosa* is included in AGM for assignment purpose. UH: undetermined hybrids, individual with relative probability < 2 but could be either F1, F2, or Backcross (BC) at probability > .95 (probability of being a purebred < .05). Inconclusive: individual with relative probability < 2 and could be either a purebred or hybrid.

## 4 DISCUSSION

### 4.1 Population structure of *Lymantria dispar* and *L. umbrosa* in their native ranges

By using genetic data together with extensive samplings from field collections, we revealed remarkably high levels of differentiation among natural populations of *Lymantria dispar* and its sister species *L. umbrosa*. Bayesian clustering and multivariate analysis suggest five major geographic groups, which are best regarded as the upper hierarchical level of population structure (Coulon et al., 2008). Four of the five groups can be further partitioned into 2–5 genetic clusters. In total, we defined 13 clusters whose relationships mirror the geography across the Northern Hemisphere when viewed from above the North Pole (Figure 4b). Our findings support the overall population structure recovered from genomic SNP and microsatellite studies (Picq et al., 2017; Wu et al., 2015; Zhang et al., 2019) and offer new insight into fine‐scale regional structuring.

The nominated subspecies *L. d. dispar* is thought to comprise populations from the western part of Eurasia with low genetic diversity (deWaard et al., 2010; Harrison, Wintermeyer, & Odell, 1983; Wu et al., 2015). In our study, it approximately corresponds to group B, which can be divided into as many as five clusters, including three from Europe. Historical work from Goldschmidt (1940) proposed two geographic races in Europe, *dispar* and *mediterranea*, for populations in northern and southern Europe, respectively. Even though the *mediterranea* race has never been formally recognized, both STRUCTURE and DAPC analyses show that moths from the Mediterranean region can be separated from other European populations (e.g., Germany), a finding that warrants re‐examination of differentiation within Europe. Additionally, it has been shown that populations from the Mediterranean region (Spain and Italy) possessed mitochondrial lineages that branched out early in the gene tree (Wu et al., 2015), which could suggest association with former glacial refugia during the Pleistocene and signal a more complicated evolutionary history than previously thought (Hewitt, 2000). Our results also showed that most *L. d. dispar* specimens possess an admixed ancestry, partially shared with individuals from Central Asia and nearby regions. In fact, populations from the latter area have the EGM‐type mitogenome but a mosaic nuclear genome transitioning from AGM to EGM (Djoumad et al., 2017; Martemyanov et al., 2019; Stewart et al., 2016; Zhao et al., 2019), a result leading to a discussion on taxonomic status of those populations (Zahiri et al., 2019).

On the far eastern end of the Eurasian continent, the evolutionary history of *L. d. asiatica*, *L. d. japonica*, and *L. umbrosa* is also intermingled. Although we demonstrated that *L. d. asiatica* and Japanese populations are distinct clusters, an admixture zone was detected along a coastal area, which encompasses northeastern China, the Korean Peninsula, and the Russian Far East (Figure 5a). This area has a continuous landscape of mixed and coniferous forest east of the Changbai Mountains with no internal geographic barrier. An extensive, admixed population with various extent of admixture likely occurs there as shown by the STRUCTURE analysis, which recovered a similar pattern based on microsatellite data (Wu et al., 2015). Among the four Japanese main islands, Kyushu and Honshu both have part of their territory stretching close to the mainland, particularly the southern tip of the Korean Peninsula. Therefore, *L. d. japonica* from the two islands likely have crossed the 200‐km‐wide Korea Strait, possibly using Tsushima Island as a stepping stone or via human‐mediated movement, and contributed to the admixture pattern observed today. Indeed, specimens with a genomic background predominantly descended from *L. d. japonica* were found at southern coastal cities of South Korea (Wu et al., 2015). A recent DNA barcoding survey of Korean *Lymantria* did not detect any *L. umbrosa* mitogenome on the peninsula (Kang, Lee, & Lee, 2015), ruling out its contribution to the establishment of the AGM admixture zone.

Interestingly, our study found a highly similar nuclear genomic background between *L. d. japonica* and *L. umbrosa*, contrasting with their consistent divergence in the mitochondrial genome (Bogdanowicz et al., 1997; Djoumad et al., 2017; Zahiri et al., 2019). The *F*_ST_ value between the two taxa is an order of magnitude smaller than values among some genetic clusters within *L. dispar* (Table 2). Separation of the two can only be recovered when analyzing lower‐level population structure. Nevertheless, this result corroborates the finding by Zhang et al. (2019) where a specimen possessing the *L. umbrosa*‐like mitogenome was grouped with *L. d. japonica*. Zhang et al. (2019) suggested that the observed discordance was caused by introgression of *L. umbrosa* mitogenome into *L. d. japonica*, a plausible hypothesis given their co‐occurrence in the northern Japanese island of Hokkaido. The two taxa can indeed be crossed under laboratory conditions but these crosses produce all‐female or all‐male broods, suggesting existence of genetic incompatibility (Goldschmidt, 1940; Higashiura et al., 2011). Although we cannot rule out the introgression hypothesis, close examination of our *L. umbrosa* specimens confirmed key morphological characteristics that match with the species diagnosis in Pogue and Schaefer (2007), such as creamy ground color of the forewing, white on the hindwing in contrast with dark margin, white legs, and tan‐colored head and thorax. Those characteristics are often used to morphologically identify the two species, since male genitalia is indistinguishable between *L. umbrosa* and *L. dispar* (Pogue & Schaefer, 2007)*.* A mutually nonexclusive hypothesis for the observed pattern would involve ancestral polymorphism, in which the nuclear genome is expected to coalescence more slowly than the mitogenome does. Therefore, both processes may account for the close relationship between the two taxa. To fully understand their taxonomic status, use of genomic SNP data, additional sampling throughout the Hokkaido Island, and re‐examination of the designated type specimens is required.

### 4.2 Genetic distinctiveness of the introduced North American population

Despite plentiful historical records on the North American population after it was introduced from Europe (Forbush & Fernald, 1896), it is yet to reach a consensus regarding its origin and distinctiveness relative to contemporary European populations. Our full and nuclear‐only datasets both identified clear differentiation between moths from the two continents, a result also supported by independent microsatellite data (Keena et al., 2008; Wu et al., 2015). The separation was not recovered by genomic SNPs generated from laboratory‐reared colonies that no longer resemble wild populations (Picq et al., 2017). However, whole nuclear genome data from a few field‐collected specimens corroborated the split between U.S. and European samples (Zhang et al., 2019). Additionally, *F*_ST_ values based on all nuclear loci between the North American and European populations were comparable to or even exceeded values among subspecies (Table 2, e.g., European *L. d. dispar* versus. *L. d. asiatica* or *L. d. japonica* versus. *L. d. asiatica*).

It has been demonstrated that founder effects in conjunction with adaptation to new environments can drive significant allele frequency changes between the founding and source population in merely tens of generations, leading to genomic differentiation (Shultz, Baker, Hill, Nolan, & Edwards, 2016; Marques, Jones, Palma, Kingsley, & Reimchen, 2018). Given the introduction history of about 150 years and a univoltine life cycle (one generation per year) in gypsy moth, the North American population should have gone through the same number of generations after the initial founding event. Therefore, differentiation very likely occurred between the source and introduced populations. Indeed, our result indicates that private nuclear and mitochondrial alleles occurring in the U.S. population are yet to be found in other populations sampled from the native range. A similar finding was reported from microsatellite data as well (Wu et al., 2015). All those lines of evidence support the unique genetic status of the North American population (i.e., an independent evolutionary lineage) distinct from contemporary European populations. Future studies are needed to understand whether such distinctiveness is a result of genetic drift, local adaptation (e.g., Friedline et al., 2019), historical admixture from multiple source populations, or a combination of those factors.

### 4.3 Geographic origin of AGM intercepted in the U.S

Using genetic data to infer the origin of biological invasions depends on the extent of population structuring in the species’ native range and whether such differentiation can be recovered by the selected markers. Previously, Picq et al. (2017) have demonstrated accurate moth assignment among laboratory‐reared colonies. It was unclear whether the assignment power would remain strong if the analysis was applied to wild populations. Our study showed that deep structure exists across the native range of *L. dispar* and the panel of 60 loci was able to recover such structure. With respect to AGM intercepted in the U.S., differentiation among East Asian populations provides the genetic basis for assigning specimens back to one of four source clusters: main China, the AGM admixture zone, *L. d. japonica*, and *L. umbrosa*. The geographic area represented by *L. d. japonica* includes all four main Japanese islands, and *L. umbrosa* is mainly found on the Hokkaido Island (Pogue & Schaefer, 2007). Resampling validation revealed high assignment success, with the exception of specimens originating from the main China cluster, which occasionally are assigned to the AGM admixture cluster.

For AGM egg masses and adults intercepted in the U.S. during 2014–2015, the majority (> 80%) originated from the AGM admixture zone. The result is strongly supported given agreement between DAPC and *assignPOP*. Consistent assignments for sibling eggs within each egg mass provide further confidence. Among the countries that the AGM admixture zone encompasses, China is the largest U.S. trading partner, while South Korea ranks 6th (International Trade Administration, 2017). Both countries have anticipation of continuing growth in trade, which could fuel more biological invasions (Seebens et al., 2015). Our results suggest that, because South Korea falls entirely within the AGM admixture zone, all its seaports should be considered potential origins of invasive AGM. On the other hand, China and Russia have a small portion of their land belonging to the AGM admixture zone, so higher risk of invasion is expected from major seaports in this region, such as the Port of Dalian in northeastern China and the Port of Vladivostok in the Russian Far East. Vigilant surveillance efforts are also necessary for Japanese seaports as well, since ~ 20% of intercepted AGM during the two sampling years were inferred to originate from Japan. To minimize the probability of AGM invasion to North America, ships and cargo departing those high‐risk ports should be strictly certified free of AGM (APHIS, 2016). Our two‐year data did not find evidence for the majority part of China (main China cluster) as the geographic origin of intercepted AGM, probably due to successful predeparture inspection and cleaning. In addition, none of the intercepted specimens is identified as *L. umbrosa*. However, this does not mean that the threat is trivial, because one *L. umbrosa* adult male was flying and later trapped in Washington during the summer of 2019 (USDA APHIS Pest Interception Database).

### 4.4 Hybridization between AGM and EGM in the Pacific Northwest

Despite predeparture inspection at Asian seaports and arrival inspection at U.S. ports of entry, a few AGM egg masses (occasionally pupae) may still be overlooked and not intercepted (Bogdanowicz, Wallner, Bell, Odell, & Harrison, 1993). Hatching larvae could disperse through ballooning into nearby forests and start to feed, and adults may directly emerge from pupae attached to cargo containers. The risk of escape is high in the Pacific Northwest, a place that falls into the hotspot category for established alien species due to high volume of international trade (Dawson et al., 2017). For example, 12 AGM adult males were trapped in Washington and Oregon in 2015, representing the largest detection of AGM adults ever documented in the U.S. (APHIS, 2016). On the other hand, inland transportation sometimes inadvertently moves EGM from its main population in the Atlantic Coast into the Pacific Northwest, thus providing mating opportunities between AGM and EGM.

Using the simulated dataset, DAPC and *assignPOP* both identified some of the 37 adult males trapped in Washington and Oregon as hybrids, most of which are likely backcrosses to EGM. Indeed, backcross hybrids are the most plausible consequence when hybridization occurs, given the abundance of EGM relative to AGM. However, among the undetermined hybrids, there was one individual that had the sum of posterior probability of membership between F1 and F2 greater than 0.95, suggesting its identity as a nonbackcross hybrid (Table S4). Compared with the simulated dataset, the laboratory dataset only represents one scenario of the hybridization process (i.e., U.S. × Japan) and thus is more conservative. It still unequivocally identified at least five specimens as hybrids. We expect that other crossing scenarios (e.g., U.S. × China or U.S. × Korea) should produce similar results, because divergence within AGM is much smaller than that between AGM and the North American population (Figure 4b, Table 2). Regardless of reference dataset or the type of test, five specimens always can be rejected as a pure AGM or EGM (combined posterior probability of membership < 0.05).

Our findings provide solid evidence for AGM‐EGM hybridization in the Pacific Northwest. This result implies that AGM arrival on cargo ships can successfully produce flying adults that have mated with the North American population, leaving genetic signatures, including alleles responsible for broader host range and stronger flight capability, in future generations. Laboratory crosses have demonstrated that, while over half of F1 females can glide over some distance, which is already an alarming observation compared to the sedentary EGM females, F2 females exhibit the full spectrum of flight capability (Keena et al., 2007). A small percentage of females from backcrosses to EGM voluntarily attempted flight (Keena et al., 2007). Introgression of Asian alleles could thus accelerate the spread of gypsy moth in North America (Keena, Wallner, Grinberg, & Cardé, 2001). In addition, although not evident among our U.S. samples (see STRUCTURE analysis), it is unknown whether hybridization and introgression have contributed to the shift of allele frequencies in the North American population, because such events may not be limited to the Pacific Northwest. Therefore, long‐term monitoring is required and immediate eradication of any potential hybrids is critical to prevent the introgression of Asian alleles into North America.

## 5 CONCLUSIONS

We characterized hierarchical genetic differentiation among worldwide populations of *Lymantria dispar* and its sister species *L. umbrosa*. Five major geographic groups were recovered, each of which, except for North America, can be further divided into multiple clusters. Population structure of gypsy moth is more complicated than previously thought and admixture is pervasive, even at species level. Our results support evolutionary distinctiveness of the introduced North American population from contemporary European populations, the historical source of the invasion. Future studies should examine whether any genetic, phenotypic, or behavioral traits have diverged in the North American population. Lower‐level structure among East Asian populations provided the basis to assign invasive individuals back to its geographic origin. The analysis can expand the capacity of current gypsy moth diagnostic assays and help track invasions of the pest around the globe. Lastly, our study suggested that invasive AGM can evade rounds of inspections, complete its development, locate an EGM mate, and produce hybrid progenies in the U.S. More effective preventative measures should be developed to address the Asian introgression into North America.

## Supporting information

Supplementary Material

## Data Availability

Data for this study are available at: https://doi.org/10.5061/dryad.xpnvx0kbr.
