# Supplementary material for: Tracking invasions of a destructive defoliator, the gypsy moth (Erebidae: Lymantria dispar): Population structure, origin of intercepted specimens, and Asian introgression into North America
Source: Evol Appl. 2020 Apr 15;13(8):2056–70. doi: 10.1111/eva.12962 (PMC7463338; doi:10.1111/eva.12962)
Supplement: Supplementary file 1 — Supplementary Material [file EVA-13-2056-s001.pdf]

Supporting Information

Figure S1. Evanno's DeltaK for the STRUCTURE analysis.

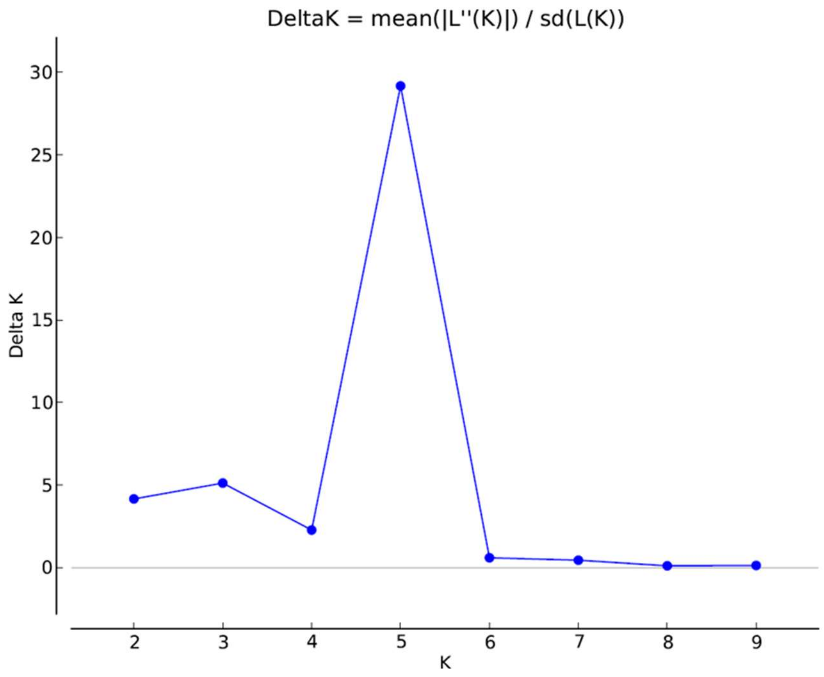

Figure S2. Structure analysis for each of the five geographic groups: delta K and posterior probability of membership.

a) North America: the ΔK method produced K = 2 or 4. However, the posterior probability does not support K > 1.

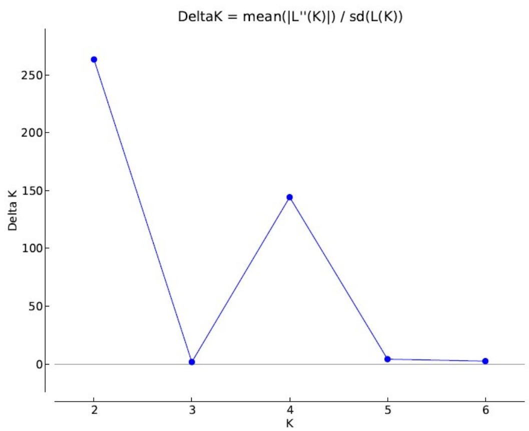

K = 1

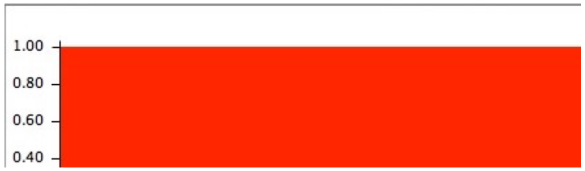

K = 2

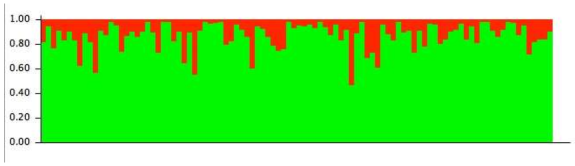

K = 4

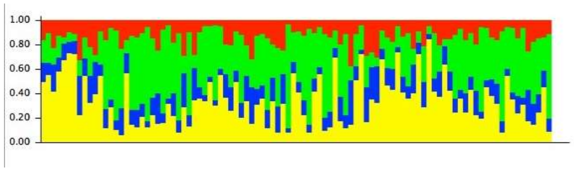

b) Europe, North Africa, Middle East

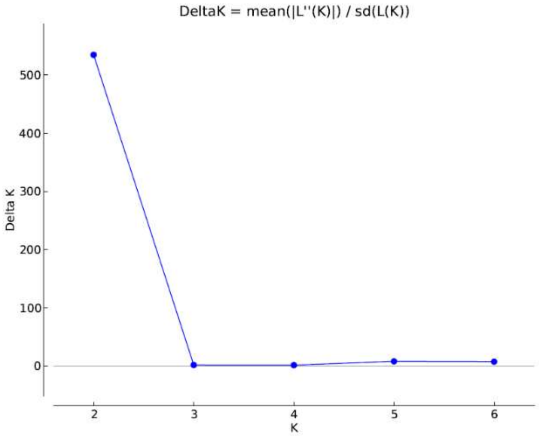

K = 2

Green: Other parts of Europe

Red: Mediterranean region (Italy, Spain, Tunisia, Syria, and a few French specimens)

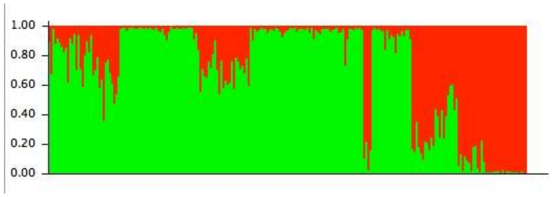

c) Central Asia, Russian Siberian, Urals

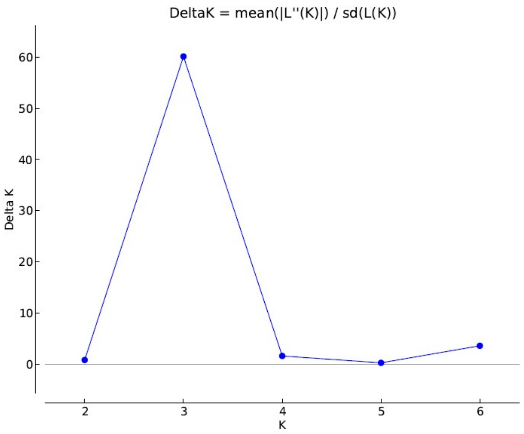

K = 3

Blue: Urals

Green: Central Asia

Red: Russia Siberia

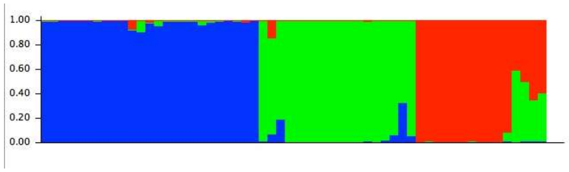

d) Continental East Asia

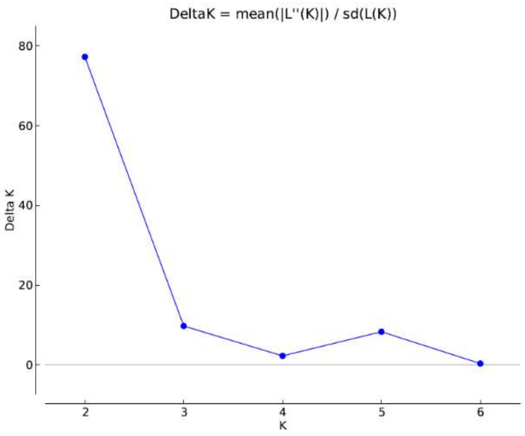

K = 2

Green: Main China

Red: northeastern China, South Korea, and the Russian Far East

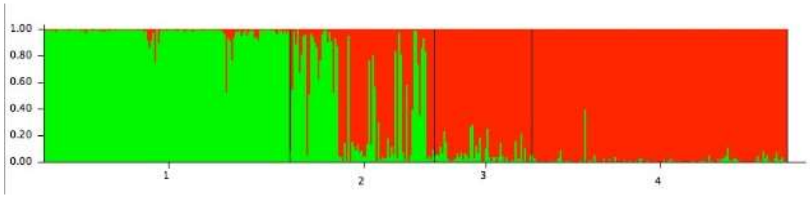

e) Japan

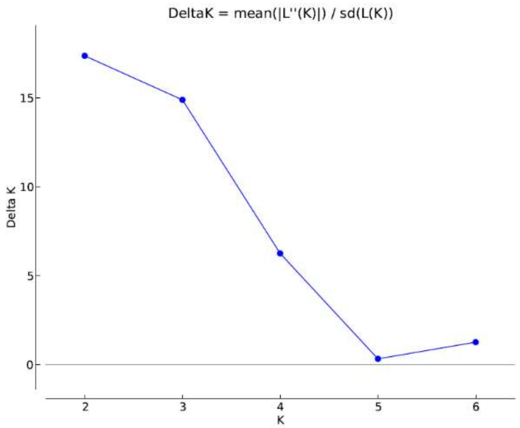

K = 2

Green: *L. d. japonica*

Red: *L. umbrosa*

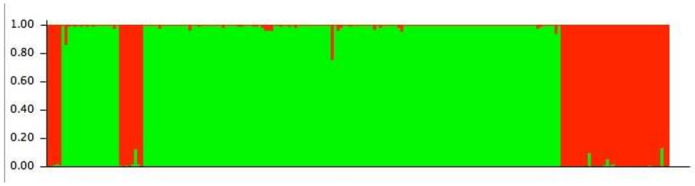

Figure S3. DAPC analysis among the world populations with nuclear and mitochondrial loci. The BIC plot suggested between 5-15 clusters. Scatter plot shows K = 5, representing the upper hierarchical level of population structure. Group A: North America; group B: Europe, North Africa, Middle East; group C: the Urals, Central Asia, Russian Siberian; group D: continental East Asia; group E: Japan.

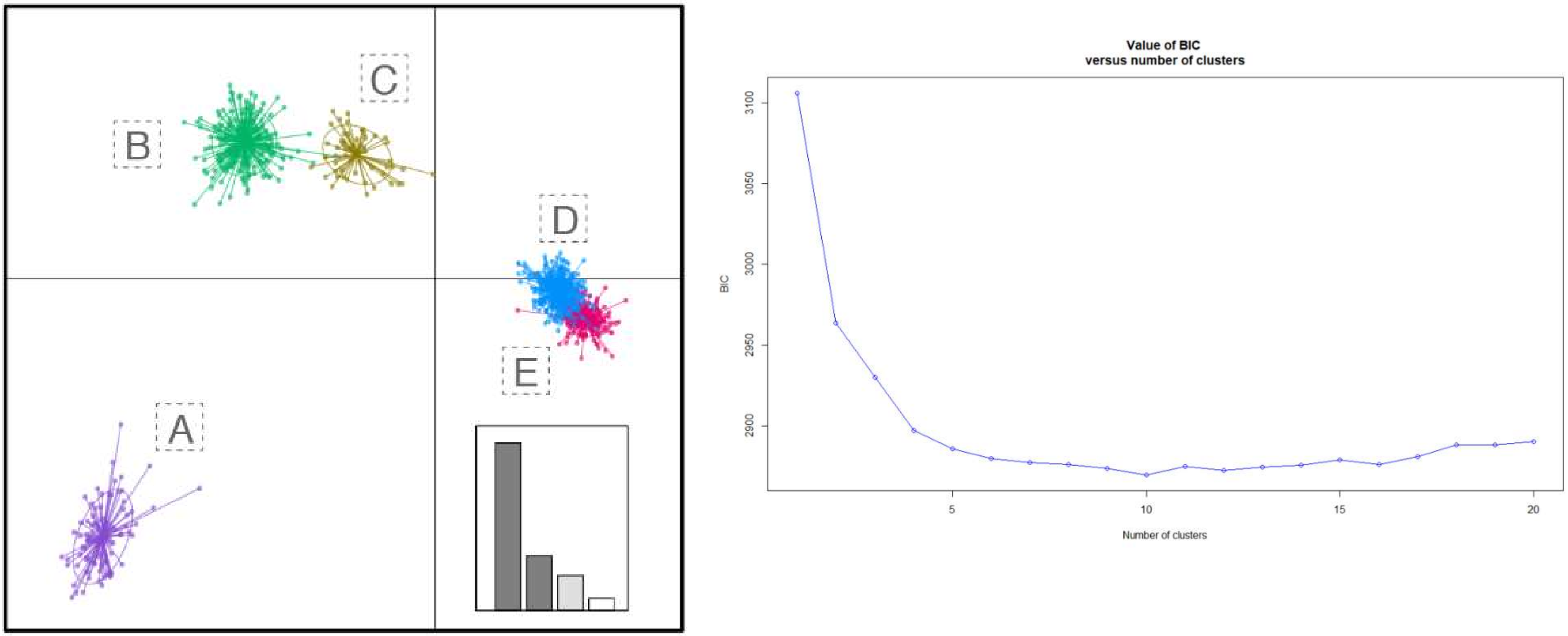

Figure S4. DAPC analysis for K = 5 with only nuclear loci. Clusters are the same as Figure S3.

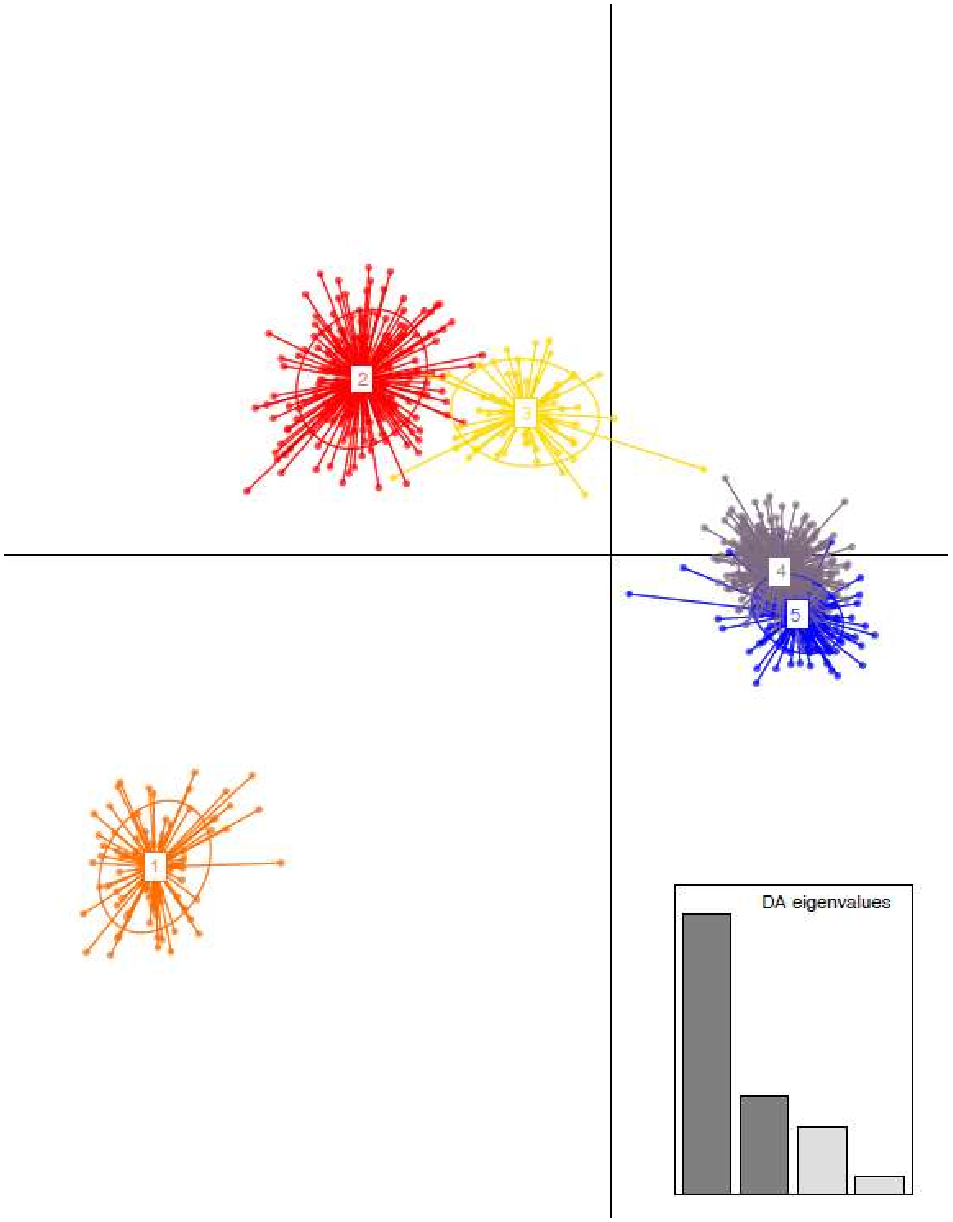

Figure S5. Bayesian Information Criterion scores and structure-like posterior probability of membership each of the five geographic groups.

a) North America

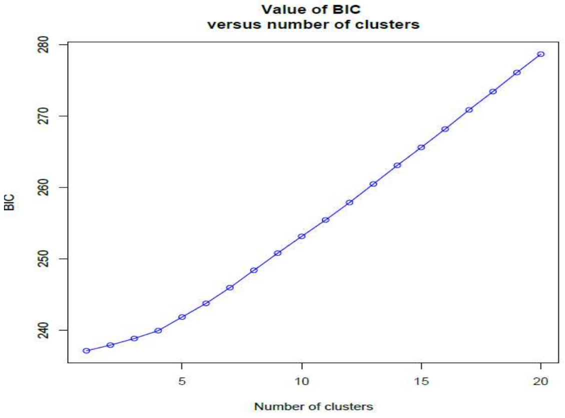

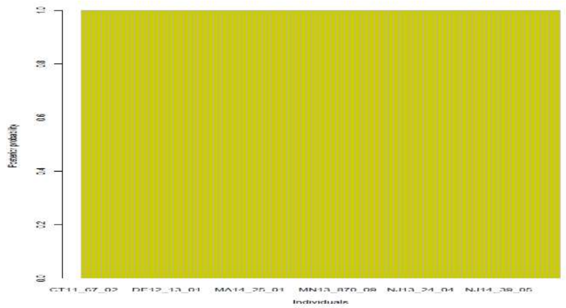

b) Europe, North Africa, Middle East

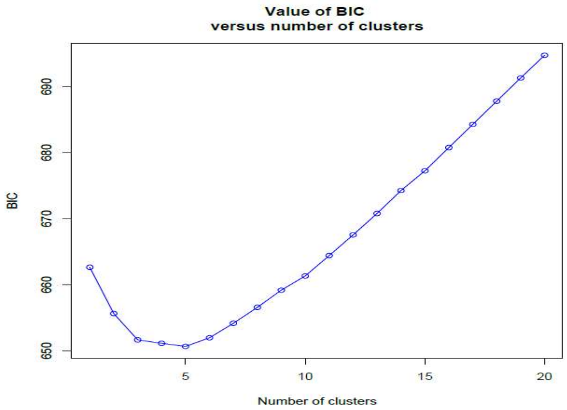

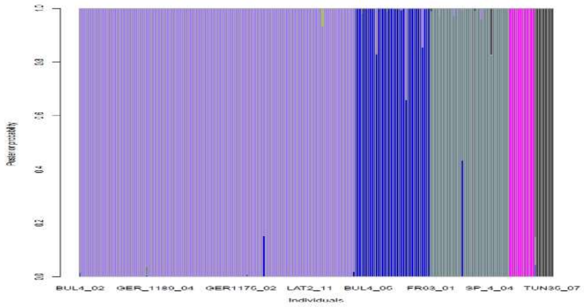

c) Central Asia, Russian Siberian, Urals

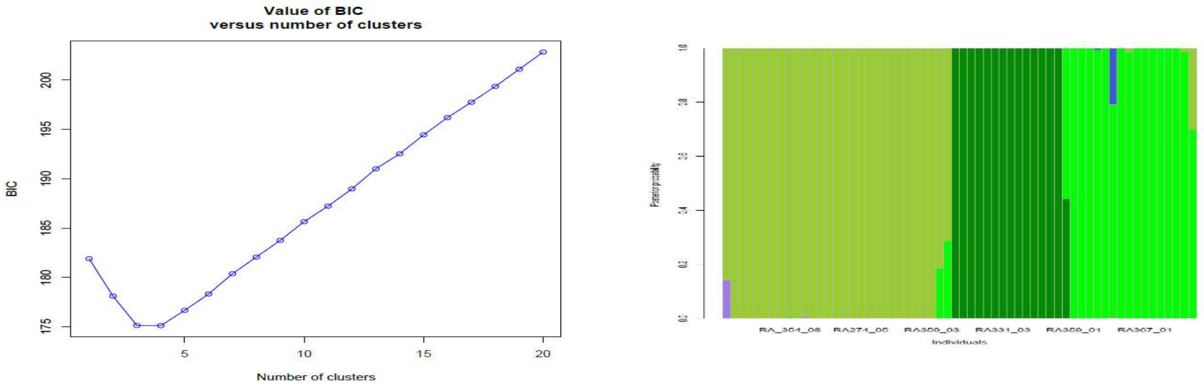

d) Continental East Asia

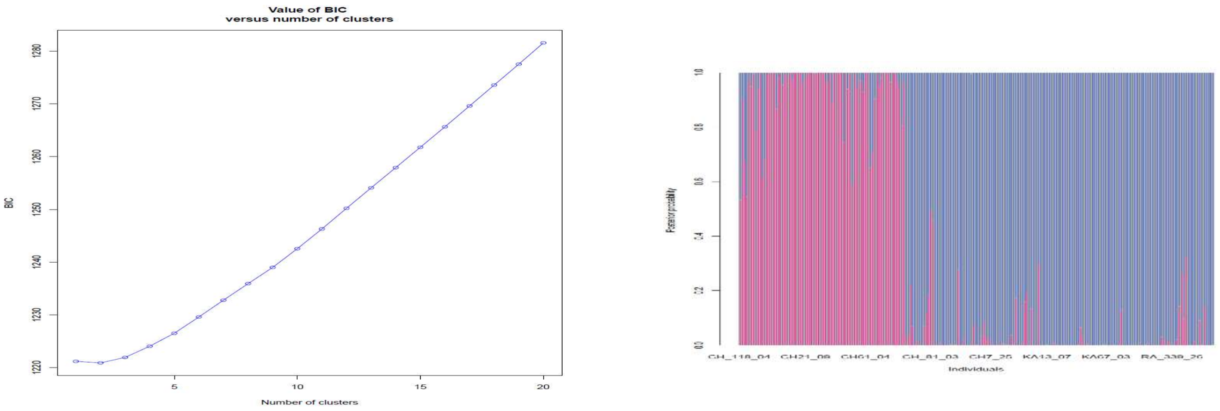

e) Japan (L. d. japonica + L. umbrosa)

i) Nuclear + mitochondrial

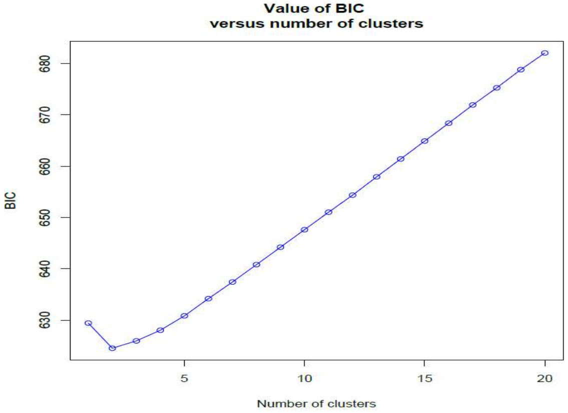

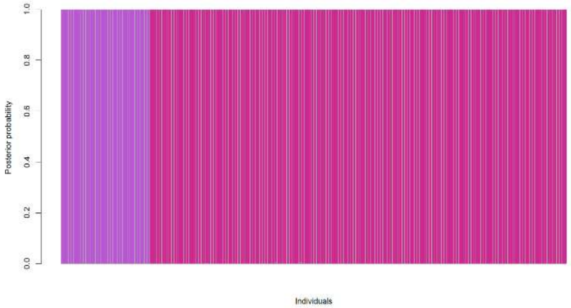

ii) Nuclear only

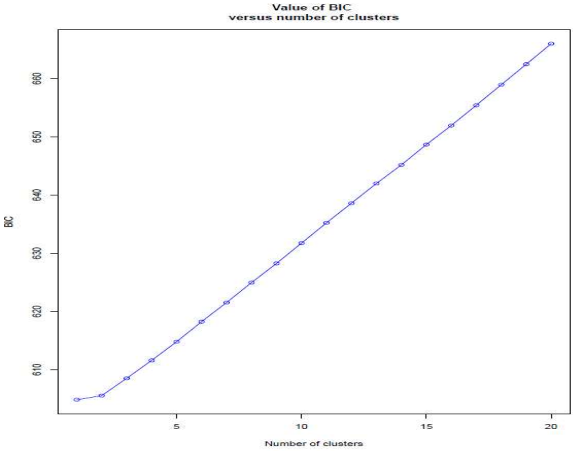

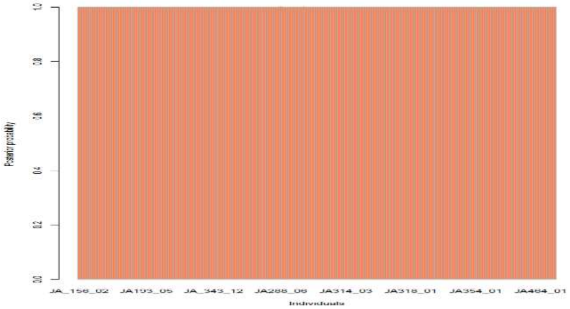

Table S1. Sampling details from 17 countries worldwide.

No.	Sample localities	Number of sample retained	Year of collection
**1**	**Main China**		
	1.1 Liu'an, Anhui	7	2013
	1.2 Tengzhou, Shandong	16	2007;2013
	1.3 Laixi, Shandong	11	1993
	1.4 Yan'an, Shaanxi	8	2013
	1.5 Tianjin	32	2007;2010
	1.6 Funing, Jiangsu	5	2011
	1.7 Jianping, Liaoning	18	2005
	1.8 Changli, Heibei	8	2007
	1.9 Beijing	8	2007
	1.10 A'ershan, Nei Mongol	8	2010
	1.11 Qiqiha'er, HeilongJiang	8	2009
	1.12 Dayi, Sichuan	2	1993
**2**	**Northeastern China**		
	2.1 Liaoyuan, Jilin	13	2008;2009
	2.2 Jiamusi, Helongjiang	8	2013
	2.3 Jiaohe, Jilin	5	2013
	2.4 Suihua, Heilongjiang	7	2008
	2.5 Daxinganlin, Heilongjiang	2	2008
	2.6 Yichun, Heilongjiang	5	2008
	2.7 Changbaishan, Jilin	11	2010
	2.8 Shenyang, Liaoning	6	1992
	2.9 Dalian, Liaoning	8	2013
	2.10 Dandong, Liaoning	8	2009
	2.11 Wafangdian, Liaoning	4	2009
**3**	**Russian Far East**		
	3.1 Vladivostok, Primorsky Krai	5	2008
	3.2 Nakhodka, Primorsky Krai	12	2008
	3.3 Olga, Primorsky Krai	21	1994
	3.4 Korfovskiy, Khabarovsk Krai	8	2014
	3.5 Lazo, Primorsky Krai	6	2014
**4**	**South Korea**		
	4.1 Ganghwa, Incheon	12	2010

	4.2 Donghae, Gangwon	26	2009;2010
	4.3 Seoul	83	2005;2007
	4.4 Busan	3	2008;2009;2010
	4.5 Incheon	4	2009
	4.6 Pohang, North Gyeongsang	5	2009
	4.7 Ulsan	3	2009
**5**	**Japan**		
	5.1 Bibai, Hokkaido	17	1992
	5.2 Sapporo, Hokkaido	23	1992
	5.3 Hakodate, Hokkaido	8	2007
	5.4 Fukuoka, Fukuoka	5	1994
	5.5 Honshu Island	48	1996
	5.6 Chiba, Chiba	11	2007
	5.7 Misawa, Aomori	6	1994
	5.8 Yokohama, Kanagawa	2	1994
	5.9 Okazaki, Aichi	2	1994
	5.10 Oyabe, Toyama	8	1994
	5.11 Kanazawa, Ishikawa	3	1994
	5.12 Soja, Okayama	6	1994
	5.13 Matsuyama, Ehime	6	1994
	5.14 Kitakyushu, Fukuoka	5	1994
	5.15 Kumomoto, Kumomoto	4	1994
	5.16 Hiroshima, Hiroshima	19	2007
	5.17 Tsukuba, Ibaraki	33	2015
**6**	**Kyrgyzstan**		
	6.1 Kara-Alma, Jalal-Abad	10	2007
**7**	**Kazakhstan**		
	7.1 Almaty, Almaty	5	2007
**8**	**Russian Siberia**		
	8.1 Novosibirsk, Novosibirsk	5	2011
	8.2 Turochak, Altai Republic	9	2014
	8.3 Gusinoye Ozero, Buryat Republic	9	2014
**9**	**Urals (Russia)**		
	9.1 Yekaterinburg, Sverdlovsk	10	2011
	9.2 Kirov, Kirov	10	1994
**10**	**Syria**		
	10.1 Latakia, Latakia	12	2008

**11**	**Bulgaria**		
	11.1 Svoge, Sofia	7	1994
	11.2 Sofia, Sofia	5	1994
	11.3 Bansko, Blagoevgrad	5	1994
	11.4 Byala, Ruse	5	1994
**12**	**Slovakia**		
	12.1 Banska Stiavnica, Banská Bystrica	12	2012
**13**	**Lithuania**		
	13.1 Kaunas, Trakai	7	1994
	13.2 Veisiejai, Lazdijai	8	1994
	13.3 Juodkrantė, Neringa	7	1994
**14**	**Latvia**		
	14.1 Grobina, Courland	5	1994
	14.2 Rucava, Courland	10	1994
**15**	**Germany**		
	15.1 Frankfurt, Hesse	6	2008
	15.2 Hohenfels, Bavaria	24	
	15.3 Ramstein-Miesenbach, Rhineland-Palatinate	11	
	15.4 Langen, Hesse	10	2005
	15.5 Dieburg, Hesse	10	2005
	15.6 Darmstadt, Hesse	11	2005
	15.7 A Germany military base	7	1993
**16**	**France**		
	16.1 Petit Landau, Alsace	15	1994
	16.2 Munchhouse, Alsace	8	1994
**17**	**Italy**		
	17.1 Cagliari, Sardinia	11	2007
	17.2 Nuoro, Sardinia	11	2006
**18**	**Spain**		
	18.1 Fresnedo, Castile and León	12	2013
**19**	**Tunisia**		
	19.1 Tabarka, Jendouba	9	1995
**20**	**US**		
	20.1 Milwaukee, Wisconsin	11	2014
	20.2 New London, Connecticut	12	2011
	20.3 Wilmington, Delaware	12	2012
	20.4 Andover, Massachusetts	12	2014

	20.5 Romulus, Michigan	12	2014
	20.6 Lake County, Minnesota	19	2013
	20.7 Linden, New Jersey	20	2014
**Total**		986	

Table S2. Primer sequences for the 60 amplicons.

Primer name	Sequences
Ldis2Fn	TCGTCGGCAGCGTCAGATGTGTATAAGAGACAGGGCTTTGGAATCAAAATGGATA
Ldis2Rn	GTCTCGTGGGCTCGGAGATGTGTATAAGAGACAGAGCCAGAATTGGGGAAAATACT
Ldis7Fn	TCGTCGGCAGCGTCAGATGTGTATAAGAGACAGGGCCTGTGTTCGTTTCTAGAGT
Ldis7Rn	GTCTCGTGGGCTCGGAGATGTGTATAAGAGACAGGAATGCTTTCAATTGGCTTAGG
Ldis47Fn	TCGTCGGCAGCGTCAGATGTGTATAAGAGACAGGAAAGTTTAAGCCCGTTCGAG
Ldis47Rn	GTCTCGTGGGCTCGGAGATGTGTATAAGAGACAGGTTATAGGGCCACACGCCTAT
Ldis72Fn	TCGTCGGCAGCGTCAGATGTGTATAAGAGACAGCGTCTTCAATGCCATCATTTAC
Ldis72Rn	GTCTCGTGGGCTCGGAGATGTGTATAAGAGACAGCCTGGCCTATATAAGTCCCACT
Ldis76Fn	TCGTCGGCAGCGTCAGATGTGTATAAGAGACAGAGGGGGTCGTATATAAGCTGAA
Ldis76Rn	GTCTCGTGGGCTCGGAGATGTGTATAAGAGACAGTTTTGTATGGCGTAAAGTGGTG
Ldis78Fn	TCGTCGGCAGCGTCAGATGTGTATAAGAGACAGCAACACTTCTAATAAGCGTGGAA
Ldis78Rn	GTCTCGTGGGCTCGGAGATGTGTATAAGAGACAGATGGATTTTAATCGCGTTTGT
Ldis98Fn	TCGTCGGCAGCGTCAGATGTGTATAAGAGACAGCATCACTTGACTAACCGACAGC
Ldis98Rn	GTCTCGTGGGCTCGGAGATGTGTATAAGAGACAGAAACGGAGATTAGAAAGAAAAATG
Ldis117Fn	TCGTCGGCAGCGTCAGATGTGTATAAGAGACAGAGGACTTACCATCGGAATACCC
Ldis117Rn	GTCTCGTGGGCTCGGAGATGTGTATAAGAGACAGTCGGTTAGACAACTTTAAATGAAGG
Ldis127Fn	TCGTCGGCAGCGTCAGATGTGTATAAGAGACAGAAAAACCTGCCATTCACTGC
Ldis127Rn	GTCTCGTGGGCTCGGAGATGTGTATAAGAGACAGTGTCCTACAAAATAATTAACAGATGC
Ldis137Fn	TCGTCGGCAGCGTCAGATGTGTATAAGAGACAGCAGGCAAGCATTAATGACACAT
Ldis137Rn	GTCTCGTGGGCTCGGAGATGTGTATAAGAGACAGCAAATGTTTTCGTTTTCCAACA
Ldis148Fn	TCGTCGGCAGCGTCAGATGTGTATAAGAGACAGGATTTAGCTGCAATGTTCAACG
Ldis148Rn	GTCTCGTGGGCTCGGAGATGTGTATAAGAGACAGATTCGAAGCCACCCACAGT
Ldis194Fn	TCGTCGGCAGCGTCAGATGTGTATAAGAGACAGGCAGGTATGTGGGAGAAATGAT
Ldis194Rn	GTCTCGTGGGCTCGGAGATGTGTATAAGAGACAGGCTGTTGTTGCTGCTGCTGTA
Ldis206Fn	TCGTCGGCAGCGTCAGATGTGTATAAGAGACAGATCCTTTGTGTCGCACAATTC
Ldis206Rn	GTCTCGTGGGCTCGGAGATGTGTATAAGAGACAGAATTACAACCAAACCGAGAACAA
Ldis224Fn	TCGTCGGCAGCGTCAGATGTGTATAAGAGACAGCAGGTACAGGGTTTTATGTCGAG
Ldis224Rn	GTCTCGTGGGCTCGGAGATGTGTATAAGAGACAGCGTTTATGAACCGATTTTGATG
Ldis306Fn	TCGTCGGCAGCGTCAGATGTGTATAAGAGACAGCGAACTCAGCGTCAGATAGATG
Ldis306Rn	GTCTCGTGGGCTCGGAGATGTGTATAAGAGACAGCGTTTACACCTGTTCTTCTACGG
Ldis341Fn	TCGTCGGCAGCGTCAGATGTGTATAAGAGACAGTACTATCGAACGATTGCAATAACC
Ldis341Rn	GTCTCGTGGGCTCGGAGATGTGTATAAGAGACAGTCTTTAGTAACACTGAATCTCGTTGC
Ldis343Fn	TCGTCGGCAGCGTCAGATGTGTATAAGAGACAGGGAATTAGTCCTTGTTGCAGGT
Ldis343Rn	GTCTCGTGGGCTCGGAGATGTGTATAAGAGACAGGCGAGTCGACTGTTGTCATAAT

Ldis413Fn	TCGTCGGCAGCGTCAGATGTGTATAAGAGACAGCGTAACACAATTAGCGCTCTTC
Ldis413Rn	GTCTCGTGGGCTCGGAGATGTGTATAAGAGACAGACGAAGGTGATATATCGCAGGT
Ldis423Fn	TCGTCGGCAGCGTCAGATGTGTATAAGAGACAGGGCCAAAATAATATCTAACTCAATGG
Ldis423Rn	GTCTCGTGGGCTCGGAGATGTGTATAAGAGACAGTGCATTTAGAAGAAATATCCAGAACA
Ldis426Fn	TCGTCGGCAGCGTCAGATGTGTATAAGAGACAGAAGGCTGCACGCCAGAAG
Ldis426Rn	GTCTCGTGGGCTCGGAGATGTGTATAAGAGACAGCCGATGTCTAAAGTCGAATTACAGG
Ldis435Fn	TCGTCGGCAGCGTCAGATGTGTATAAGAGACAGTCCTGGTTCACCTTTATCACCT
Ldis435Rn	GTCTCGTGGGCTCGGAGATGTGTATAAGAGACAGTGGAGTTAATATTGTTCCATTCTGA
Ldis467Fn	TCGTCGGCAGCGTCAGATGTGTATAAGAGACAGGAGAAAGGAATGGGTTGGTAAA
Ldis467Rn	GTCTCGTGGGCTCGGAGATGTGTATAAGAGACAGTCACATACTAACAGGGCAAGTGA
Ldis479Fn	TCGTCGGCAGCGTCAGATGTGTATAAGAGACAGGGGAATGGGCTGTAATACATAAA
Ldis479Rn	GTCTCGTGGGCTCGGAGATGTGTATAAGAGACAGTGGAATTGATTCATTCACATATACAA
Ldis32Fn	TCGTCGGCAGCGTCAGATGTGTATAAGAGACAGAGGTCCCACCGGAGAGTTC
Ldis32Rn	GTCTCGTGGGCTCGGAGATGTGTATAAGAGACAGAAAAATCCGAACGGAACATTTA
Ldis1128Fn	TCGTCGGCAGCGTCAGATGTGTATAAGAGACAGCTTCTGCCGTCCAACAGTATT
Ldis1128Rn	GTCTCGTGGGCTCGGAGATGTGTATAAGAGACAGGTTGGTTCGTTGAGCGATGT
Ldis1426Fn	TCGTCGGCAGCGTCAGATGTGTATAAGAGACAGCATGAAGATGAAACGGTCAGAA
Ldis1426Rn	GTCTCGTGGGCTCGGAGATGTGTATAAGAGACAGTTTAATGTGAACGACATCACAGC
Ldis1486Fn	TCGTCGGCAGCGTCAGATGTGTATAAGAGACAGAAACTGACAGCAGCGTGACTTA
Ldis1486Rn	GTCTCGTGGGCTCGGAGATGTGTATAAGAGACAGTGTGTAGCAGTGTCCACGAGAT
Ldis1449Fn	TCGTCGGCAGCGTCAGATGTGTATAAGAGACAGCACCTGAACCGTTACACTCATT
Ldis1449Rn	GTCTCGTGGGCTCGGAGATGTGTATAAGAGACAGACCACAACGTGTATCAACTTCC
Ldis1432Fn	TCGTCGGCAGCGTCAGATGTGTATAAGAGACAGTTGATCTGGCCAATTTTAAGGT
Ldis1432Rn	GTCTCGTGGGCTCGGAGATGTGTATAAGAGACAGCGACAGCTGATGAACCTGTCTA
Ldis1196Fn	TCGTCGGCAGCGTCAGATGTGTATAAGAGACAGCCACATAACGGGCCTAGAATAG
Ldis1196Rn	GTCTCGTGGGCTCGGAGATGTGTATAAGAGACAGATTTCAGGAAACGAGCTTTCAC
Ldis1139Fn	TCGTCGGCAGCGTCAGATGTGTATAAGAGACAGAATAAAGGCGAGCTGGATACG
Ldis1139Rn	GTCTCGTGGGCTCGGAGATGTGTATAAGAGACAGTTGGGCGTTACATAACTCTTCG
Ldis505Fn	TCGTCGGCAGCGTCAGATGTGTATAAGAGACAGCTGTGGTCCAACAAGAGTTGAG
Ldis505Rn	GTCTCGTGGGCTCGGAGATGTGTATAAGAGACAGAATAAGCAGACAAGCATTGACG
Ldis524Fn	TCGTCGGCAGCGTCAGATGTGTATAAGAGACAGGGAGGCGCTAACGTGTAAACTA
Ldis524Rn	GTCTCGTGGGCTCGGAGATGTGTATAAGAGACAGGCGTATTATGTTTCGAAGTCGTC
Ldis583Fn	TCGTCGGCAGCGTCAGATGTGTATAAGAGACAGCTGCCGGATCTTGTATCAGTTT
Ldis583Rn	GTCTCGTGGGCTCGGAGATGTGTATAAGAGACAGGCTTCAACTGCATAACACTGTCT
Ldis617Fn	TCGTCGGCAGCGTCAGATGTGTATAAGAGACAGCAGGTGTAGTGGAAGATGGAAA
Ldis617Rn	GTCTCGTGGGCTCGGAGATGTGTATAAGAGACAGTTTGGACAACCAAAACTGAAAA
Ldis641Fn	TCGTCGGCAGCGTCAGATGTGTATAAGAGACAGAATGATTTAGCTGCAATGTTCAA

Ldis641Rn	GTCTCGTGGGCTCGGAGATGTGTATAAGAGACAGACTATGCCATCAGTGCAGTCAG
Ldis649Fn	TCGTCGGCAGCGTCAGATGTGTATAAGAGACAGTTTGAAGAGAATCTCAAGTTTTGC
Ldis649Rn	GTCTCGTGGGCTCGGAGATGTGTATAAGAGACAGTTATTTCAGCCCTTGTTCAGGT
Ldis672Fn	TCGTCGGCAGCGTCAGATGTGTATAAGAGACAGGCTGAGGGATCACCAGGATAAT
Ldis672Rn	GTCTCGTGGGCTCGGAGATGTGTATAAGAGACAGTTTTTACGATTTTGTTCGCACTT
Ldis678Fn	TCGTCGGCAGCGTCAGATGTGTATAAGAGACAGGTTTGCATCTGTACCTTCACCA
Ldis678Rn	GTCTCGTGGGCTCGGAGATGTGTATAAGAGACAGGTGACACATGGGTCTCTCGTC
Ldis721Fn	TCGTCGGCAGCGTCAGATGTGTATAAGAGACAGGGGTACAGTCCTACTCCAGTTAAGG
Ldis721Rn	GTCTCGTGGGCTCGGAGATGTGTATAAGAGACAGGGTGACGGAGCAAGAATACAAT
Ldis731Fn	TCGTCGGCAGCGTCAGATGTGTATAAGAGACAGTGGATGTACGTACTACGCTGCT
Ldis731Rn	GTCTCGTGGGCTCGGAGATGTGTATAAGAGACAGCGTGCTAATTCTTCGTGCTATG
Ldis749Fn	TCGTCGGCAGCGTCAGATGTGTATAAGAGACAGAGGAGCTCAGCGATTGCTTT
Ldis749Rn	GTCTCGTGGGCTCGGAGATGTGTATAAGAGACAGTGTCAGGGAGAGTGAGAGAGAA
Ldis1148Fn	TCGTCGGCAGCGTCAGATGTGTATAAGAGACAGAGGATTCCATTTCGAGTTTTCA
Ldis1148Rn	GTCTCGTGGGCTCGGAGATGTGTATAAGAGACAGCACTTCAGCGTGCTATTTCCTC
Ldis1240Fn	TCGTCGGCAGCGTCAGATGTGTATAAGAGACAGGTTCATGCCAGTCAACTCTCTG
Ldis1240Rn	GTCTCGTGGGCTCGGAGATGTGTATAAGAGACAGGCCCCTACTACACCACTCTGAT
Ldis1282Fn	TCGTCGGCAGCGTCAGATGTGTATAAGAGACAGAGTCAAGGAATCGGTACCAGTG
Ldis1282Rn	GTCTCGTGGGCTCGGAGATGTGTATAAGAGACAGGGCAAGATAGAGTGCTGAATTG
Ldis1335Fn	TCGTCGGCAGCGTCAGATGTGTATAAGAGACAGAGTCTCGCGTCTTGCATTG
Ldis1335Rn	GTCTCGTGGGCTCGGAGATGTGTATAAGAGACAGTAATTCTGAAAAGCCAACACCA
Ldis1336Fn	TCGTCGGCAGCGTCAGATGTGTATAAGAGACAGGGTCGCCATTCTACGTTACTGT
Ldis1336Rn	GTCTCGTGGGCTCGGAGATGTGTATAAGAGACAGTGTATGTATCCGAACTAGCCGTA
Ldis1354Fn	TCGTCGGCAGCGTCAGATGTGTATAAGAGACAGATAAAGGCGAGCTGAATACGAG
Ldis1354Rn	GTCTCGTGGGCTCGGAGATGTGTATAAGAGACAGGGTGGGTCCAACTTATAGCCTAC
Ldis1375Fn	TCGTCGGCAGCGTCAGATGTGTATAAGAGACAGCAATATTAAACATGCATCACTTTGT
Ldis1375Rn	GTCTCGTGGGCTCGGAGATGTGTATAAGAGACAGATGTGGAAATTCAGCAGGTAGG
Ldis1400Fn	TCGTCGGCAGCGTCAGATGTGTATAAGAGACAGGTGCAGGGTGTGCGTAAAA
Ldis1400Rn	GTCTCGTGGGCTCGGAGATGTGTATAAGAGACAGGCTTCTCTTCTTATTGCCTTGG
Ldis1415Fn	TCGTCGGCAGCGTCAGATGTGTATAAGAGACAGAAAAAGGGACTACCGATTTTCA
Ldis1415Rn	GTCTCGTGGGCTCGGAGATGTGTATAAGAGACAGACATTATAAAAATTTTCGCGTGGT
Ldis1436Fn	TCGTCGGCAGCGTCAGATGTGTATAAGAGACAGGCATGCACAATGGAGAGC
Ldis1436Rn	GTCTCGTGGGCTCGGAGATGTGTATAAGAGACAGCAGTGGGTGAAATTGAGCATAG
Ldis1467Fn	TCGTCGGCAGCGTCAGATGTGTATAAGAGACAGGAGGCTAGGGACAGTGGAAG
Ldis1467Rn	GTCTCGTGGGCTCGGAGATGTGTATAAGAGACAGTTCCTCTGGGTTGCTGAATAAT
Ldis1483Fn	TCGTCGGCAGCGTCAGATGTGTATAAGAGACAGTTAGGAGAATTAGGAGAGTTGAGGA
Ldis1483Rn	GTCTCGTGGGCTCGGAGATGTGTATAAGAGACAGAAGTAACCAGAGTAAGTCAGCGAGT

Ldis1192Fn	TCGTCGGCAGCGTCAGATGTGTATAAGAGACAGGTAGGATTCGGTCGCCATTAGT
Ldis1192Rn	GTCTCGTGGGCTCGGAGATGTGTATAAGAGACAGAATTCTGTCAGCAGTCCTCTCG
LdiscytbFn	TCGTCGGCAGCGTCAGATGTGTATAAGAGACAGAGCCCGTCCTGTTGAAGAC
LdiscytbRn	GTCTCGTGGGCTCGGAGATGTGTATAAGAGACAGAGAGGGGGATTTGCTTTGA
LdisND4Fn	TCGTCGGCAGCGTCAGATGTGTATAAGAGACAGACTATTCTTATATGGGCAACAGATG
LdisND4Rn	GTCTCGTGGGCTCGGAGATGTGTATAAGAGACAGAACGTATTTTAGCTGGGATGTATC
LdisND2Fn	TCGTCGGCAGCGTCAGATGTGTATAAGAGACAGTGCCTGATTAAAGGATTATTCTGA
LdisND2Rn	GTCTCGTGGGCTCGGAGATGTGTATAAGAGACAGCAAAAATGAAAGGGGGATGA
LdisCOIIIFn	TCGTCGGCAGCGTCAGATGTGTATAAGAGACAGTAACAAAAGGATTACGCTGAGGTA
LdisCOIIIRn	GTCTCGTGGGCTCGGAGATGTGTATAAGAGACAGATATCATGCTGCGGCTTCA
Ldis16SFn	TCGTCGGCAGCGTCAGATGTGTATAAGAGACAGAAATTACGCTGTTATCCCTAAGGT
Ldis16SRn	GTCTCGTGGGCTCGGAGATGTGTATAAGAGACAGCGGCAAAATTTTATATTCACTTGT

Table S3. A) Assignment probabilities for intercepted individual AGM eggs and adults based on DAPC. Results from *assignPOP* was highly similar. B) Inferred origin for AGM egg masses and adults.

A)

Specimens	Main China	Mixture zone	Japan	L. umbrosa
AK14_1_01	0.01	0.99	0	0
AK14_2_03	0.04	0.96	0	0
AK14_2_04	0	1	0	0
AK14_4_02	0.03	0.97	0	0
AK14_4_03	0	1	0	0
AK14_4_04	0.01	0.99	0	0
AK141_03	0	1	0	0
AK141_04	0	1	0	0
AK142_01	0.03	0.97	0	0
AK143_03	0	1	0	0
AK143_04	0	1	0	0
AK144_01	0.01	0.99	0	0
CA14_27_02	0	1	0	0
CA14_27_03	0	1	0	0
CA14_29_02	0	1	0	0
CA14_29_04	0	1	0	0
CA14_31_02	0	0.97	0.03	0
CA14_31_04	0.02	0.98	0	0
CA14_32_01	0.01	0.99	0	0
CA14_43_02	0.02	0.98	0	0
CA14_43_03	0	1	0	0
CA14_43_04	0.01	0.99	0	0
CA1427_01	0.53	0.47	0	0
CA1428_03	0	1	0	0
CA1429_01	0	1	0	0
CA1430_03	0	1	0	0
CA1430_04	0	1	0	0
CA1431_01	0	1	0	0
CA1432_03	0	1	0	0
CA1432_04	0	1	0	0

CA1439_03	0	1	0	0
CA1439_04	0	1	0	0
CA1443_01	0.01	0.99	0	0
CA1444_03	0	1	0	0
CA1444_04	0	1	0	0
CA1448_01	0	1	0	0
CA1449_03	0.35	0.65	0	0
CA1449_04	0.01	0.99	0	0
CA1459_01	0	0	1	0
CA1459_02	0	0	1	0
CA1459_03	0	0	1	0
CA1459_04	0	0	1	0
CA1459_05	0	0	1	0
CA1459_06	0	0	1	0
CA1459_07	0	0	1	0
CA1459_08	0	0	1	0
OR14105_02	0	1	0	0
OR14105_03	0	1	0	0
OR14105_04	0	0.96	0.04	0
OR14106_01	0	1	0	0
OR14106_02	0.01	0.99	0	0
OR14106_03	0	1	0	0
OR14106_04	0.05	0.95	0	0
OR14107_01	0.01	0.99	0	0
OR14107_02	0.01	0.99	0	0
OR14107_03	0.03	0.97	0	0
OR14107_04	0.03	0.97	0	0
OR14108_01	0.03	0.97	0	0
OR14108_02	0	1	0	0
OR14108_03	0	1	0	0
OR14108_04	0	1	0	0
OR1425_01	0	0	1	0
OR1425_02	0	0	1	0
OR1425_03	0	0	1	0
OR1425_04	0	0	1	0
OR1426_01	0	0	1	0
OR1426_02	0	0	1	0

OR1426_03	0	0	1	0
OR1426_04	0	0	1	0
OR1427_01	0	1	0	0
OR1427_02	0	0	1	0
OR1427_03	0	0	1	0
OR1427_04	0	0	1	0
TX1411_01	0	0	1	0
TX1411_02	0	0	1	0
TX1411_03	0	0	1	0
WA143_01	0.01	0.99	0	0
WA143_02	0.02	0.98	0	0
WA143_03	0	1	0	0
WA143_04	0	1	0	0
WA144_01	0	1	0	0
WA144_02	0.01	0.99	0	0
WA144_03	0	1	0	0
WA144_04	0.01	0.99	0	0
SC141_01a	0	1	0	0
OR158_01	0	1	0	0
WA1513_01	0.01	0.99	0	0
WA15_19_01	0	1	0	0
WA1520_01	0	0	1	0

B)

	Intercepted Life Stage	Intercepted Port or Location	Inferred Origin
AK14_1	Egg Mass	Juneau, AK	Mixture Zone
AK14_2	Egg Mass	Juneau, AK	Mixture Zone
AK14_3	Egg Mass	Juneau, AK	Mixture Zone
AK14_4	Egg Mass	Juneau, AK	Mixture Zone
CA14_27	Egg Mass	Long Beach, CA	Mixture Zone
CA14_28	Egg Mass	Long Beach, CA	Mixture Zone
CA14_29	Egg Mass	Long Beach, CA	Mixture Zone
CA14_30	Egg Mass	Long Beach, CA	Mixture Zone
CA14_31	Egg Mass	Long Beach, CA	Mixture Zone
CA14_32	Egg Mass	Long Beach, CA	Mixture Zone

CA14_39	Egg Mass	Long Beach, CA	Mixture Zone
CA14_43	Egg Mass	Long Beach, CA	Mixture Zone
CA14_44	Egg Mass	Long Beach, CA	Mixture Zone
CA14_48	Egg Mass	Long Beach, CA	Mixture Zone
CA14_49	Egg Mass	Long Beach, CA	Mixture Zone
CA14_59	Egg Mass	Long Beach, CA	Japan
OR14_25	Egg Mass	Portland, OR	Japan
OR14_26	Egg Mass	Portland, OR	Japan
OR14_27	Egg Mass	Portland, OR	Conflicting results
OR14_105	Egg Mass	Portland, OR	Mixture Zone
OR14_106	Egg Mass	Portland, OR	Mixture Zone
OR14_107	Egg Mass	Portland, OR	Mixture Zone
OR14_108	Egg Mass	Portland, OR	Mixture Zone
TX14_11	Egg Mass	Houston, TX	Japan
WA14_3	Egg Mass	Seattle, WA	Mixture Zone
WA14_4	Egg Mass	Seattle, WA	Mixture Zone
SC14_1_01	Adult	North Charleston, SC	Mixture Zone
OR15_8_01	Adult	Portland, OR	Mixture Zone
WA15_13_01	Adult	Tacoma, WA	Mixture Zone
WA15_19_01	Adult	Kent, WA	Mixture Zone
WA15_20_01	Adult	Tacoma, WA	Japan

Table S4. Specimen ID, trap number, collection site, and posterior probabilities of membership for Oregon and Washington specimens from DAPC (before slash) and *assignPOP* (after slash) based on the simulated dataset. The five specimens highlighted in red were always assigned as hybrids regardless of dataset (simulated vs. laboratory) or test methods.

	Trap #	County	Collection Site	AGM	US	F1	F2	BC1
OR1512_01	67-46425	Washington	Forest Grove, OR	0/0	0.44/0.46	0/0.01	0/0.02	0.56/0.51
OR154_04	33-7049	Josephine	Grants Pass, OR	0/0	0/0.04	0/0.01	0/0.03	1/0.92
OR158_01	51-50817	Multnomah	Portland, OR	1/1	0/0	0/0	0/0	0/0
WA14_10_01	1503-1124	King	Seattle, WA	0/0	1/0.99	0/0	00	0/0.1
WA14_23_01	1283-069	King	Seattle, WA	0/0	1/0.97	0/0	0/0	0/0.03
WA14_24_01	1375-119	King	Seattle, WA	0/0	1/1	0/0	0/0	0/0
WA14_25_01	1547-796	Whatcom	Bellingham, WA	0/0	1/0.97	0/0	0/0	0/0.03
WA14_26_01	1551-1043	Clark	Yacolt, WA	0/0	1/0.98	0/0	0/0	0/0.02
WA14_27_01	1503-626	King	Seattle, WA	0/0	1/0.97	0/0	0/0	0/0.03
WA14_28_01	1503-1110	King	Seattle, WA	0/0	1/0.99	0/0	0/0	0/0.01
WA14_5_01	1528-032	Jefferson	Port Townsend, WA	0/0	1/0.98	0/0	0/0	0/0.02
WA14_6_01	1503-498	King	Seattle, WA	0/0	1/1	0/0	0/0	0/0
WA14_7_01	1503-498	King	Seattle, WA	0/0	1/0.97	0/0	0/0	0/0.03
WA14_8_01	1503-1117	King	Seattle, WA	0/0	0.99/0.97	0/0	0/0	0.01/0.03
WA14_9_01	983-759	Clallam	Neah Bay, WA	0/0	1/0.97	0/0	0/0	0/0.03
WA15_10_01	631-005	King	Seattle, WA	0/0	0/0	0/0.5	0/0.5	1/0
WA15_19_01	1560-126	King	Kent, WA	0.65/0.98	0/0	0.12/0.01	0.24/0.01	0/0
WA15_32_03	1572-229	Thurston	Olympia, WA	0/0	0/0.02	0/0.03	0/0.07	1/0.88
WA1513_01	1558-049	Pierce	Tacoma, WA	1/1	0/0	0/0	0/0	0/0
WA1515_01	631-003	King	Seattle, WA	0/0	0.18/0.06	0/0.01	0/0.02	0.82/0.91
WA152_01	1511-121	King	Seattle, WA	0/0	0.14/0.19	0/0.01	0/0.02	0.86/0.78
WA1520_01	1558-226	Pierce	Tacoma, WA	1/1	0/0	0/0	0/0	0/0
WA1521_01	1511-140	King	Seattle, WA	0/0	1/0.91	0/0.01	0/0.01	0/0.07
WA1522_01	1363-074	Kitsap	Port Orchard, WA	0/0	0.11/0.45	0/0.01	0/0.02	0.89/0.52
WA1527_01	1511-142	King	Seattle, WA	0/0.02	0/0	0/0.15	0/0.36	1/0.37
WA1528_01	1528-236	Jefferson	Port Townsend, WA	0/0.01	0/0.07	0/0.16	0/0.36	1/0.40
WA153_01	1511-142	King	Seattle, WA	0/0	0.3/0.64	0/0.01	0/0.02	0.7/0.33
WA1532_01	1572-229	Thurston	Olympia, WA	0/0	1/0.95	0/0	0/0.01	0/0.04
WA1532_02	1572-229	Thurston	Olympia, WA	0/0	1/0.92	0/0.01	0/0.01	0/0.06
WA154_01	1511-143	King	Seattle, WA	0/0	0/0.04	0.49/0.35	0.39/0.46	0.11/0.15

WA155_01	631-004	King	Seattle, WA	0/0	0/0.02	0.13/0.37	0.1/0.45	0.78/0.16
WA156_01	631-002	King	Seattle, WA	0/0.01	0/0.05	0.01/0.09	0.01/0.23	0.98/0.62
WA156_03	631-002	King	Seattle, WA	0/0	0.07/0.21	0/0.01	0/0.02	0.93/0.76
WA157_01	631-005	King	Seattle, WA	0/0	0.11/0.14	0/0.01	0/0.01	0.89/0.84
WA157_02	631-005	King	Seattle, WA	0/0	0.66/0.38	0/0.01	0/0.02	0.34/0.59
WA158_01	631-003	King	Seattle, WA	0/0	0/0.02	0.33/0.41	0.21/0.47	0.46/0.10
WA159_01	1511-142	King	Seattle, WA	0/0	0/0.02	0.53/0.45	0.44/0.50	0.02/0.03
